# All Solid Lithium Metal‐Polymer Battery End‐of‐Life: an Investigation of Symmetric, Battery, and Bilayer Cells

**DOI:** 10.1002/advs.202520395

**Published:** 2026-03-12

**Authors:** Lucile Magnier, Didier Devaux, Jérôme Adrien, Ce Xiao, Philippe Dumaz, Margaud Lécuyer, Marc Deschamps, Eric Maire, Renaud Bouchet

**Affiliations:** ^1^ INSA Lyon, CNRS UMR 5510 MATEIS Villeurbanne France; ^2^ Univ. Grenoble Alpes, Univ. Savoie Mont Blanc, CNRS, Grenoble INP LEPMI Grenoble France; ^3^ Blue Solutions Ergué Gabéric Quimper France

**Keywords:** battery end‐of‐life, electrochemical measurement, ${\mathrm{LiFePO}}_{4}$, lithium metal, polymer electrolyte, short‐circuit, X‐ray tomography

## Abstract

Li metal as a negative electrode material is very promising to reach higher energy density batteries beyond Li‐ion. Batteries using a solid polymer electrolyte (SPE), such as poly(ethylene oxide), can cycle over several thousand cycles, but their end‐of‐life mechanism remains unclear. Failure modes may originate from both positive and/or negative active electrode materials and at the active material/electrolyte interfaces. For the Li metal negative electrode, X‐ray computed tomography (XRCT) is a powerful and accurate tool to visualize and quantify the heterogeneity of the Li electrodeposits induced by the oxidation (pitting) and reduction (stripping) electrochemical steps. This work investigates the impact of cycling on symmetric cells and reveals the direct link between the heterogeneities induced by Li microstructure and the electrochemical signature of cell shortcut. To go further in the investigation, full batteries and bilayer positive cells were analyzed using the same approach, but major differences were highlighted compared to symmetric cells. This work highlights the importance of studying full batteries to understand the behavior of Li metal during long‐term cycling. A failure mechanism occurring in Li metal batteries is proposed in light of these new findings.

## Graphical abstract

In this study, the evolution of the lithium (Li)/polymer interface is compared in a realistic pouch cell configuration, either in Li/Li symmetric cell or in full Li/${\mathrm{LiFePO}}_{4}$ batteries thanks to X‐ray micro‐tomography. While Li/Li symmetric cells are the reference cell for probing the interface stability, results demonstrated that the end‐of‐life and limiting processes are completely different in a full battery.

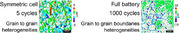

## 1 Introduction

To meet the challenge of electric vehicle democratization, the development of efficient, cheap and safe batteries is necessary. Lithium (Li) metal is considered as one of the most promising negative electrode material thanks to its low electrochemical potential and high theoretical capacity [1, 2, 3]. In contact with an electrolyte, Li reactivity induces the formation of a passive surface layer, called Solid Electrolyte Interphase (SEI) [4], whose composition varies according to the electrolyte [5, 6, 7]. During battery cycling, alternatively Li metal is oxidized in ${\mathrm{Li}}^{+}$ ions (stripping‐discharge) and ${\mathrm{Li}}^{+}$ ions are reduced in Li metal (plating‐charge). During the later, the electrodeposition on the Li metal surface generally leads to heterogeneous nucleation (named abusively dendrites), which grows until it reaches the positive electrode, short‐circuiting the cell [8]. In the early research on Li metal battery, this phenomenon slowed down their commercialization, because of safety hazards [9, 10, 11]. Numerous publications have investigated this heterogeneous deposition, which depends on several parameters such as SEI composition and microstructure [12, 13, 14], temperature [15] and current density [16, 17, 18]. Li stripping is also a heterogeneous process, which leads to surface pitting [19, 20], but is less studied than plating inducing a knowledge gap [21]. Key parameters known to impact both stripping and plating are: Li microstructure, grain boundaries [22, 23]. surface topology [24, 25], and surface chemistry [26].

To improve battery lifetime, a possible approach is to use a mechanical barrier to prevent dendrite from growing through the electrolyte. Monroe and Newman have shown theoretically that when using a Solid Polymer Electrolyte (SPE), the surface roughness is suppressed when its shear modulus is greater than twice that of Li metal [27]. Using block copolymers PS‐POE laden with LiTFSi, Hallinan et al. have experimentally verified the result of Newman and Monroe [28]. The use of a SPE effectively slows down dendrite growth through the electrolyte but stripping and plating remain heterogeneous leading to limited battery cycle life [29, 30]. Numerous studies carried out by Balsara's group have revealed heterogeneities linked to the presence of impurities in the Li electrode [23, 31]. These electronically insulating inclusions lead to preferential deposits around them, resulting in large local current density heterogeneities [32], which leads after cycling to the formation of globular Li structure surrounding the impurity and spanning into the electrolyte. These globules ultimately shorts the cells, and reducing their quantity increases the cycle‐life of symmetric cells [33]. Thus, these inclusions play a key role in Li metal/polymer battery cycling.

${\mathrm{LiFePO}}_{4}$/SPE/Li‐metal batteries, commercialized by Bolloré (Blue Solutions), have an energy density of 180 Wh.${\mathrm{kg}}^{-1}$ and 80 % capacity retention after 1300 cycles [34]. Hovington et al. also demonstrated the high performances of ${\mathrm{LiFePO}}_{4}$/SPE/Li‐metal batteries with a capacity maintained at 130 mAh.$g^{-1}$ after 1400 cycles [35]. Hallinan et al. studied the end‐of‐life of these batteries by electrochemical measurement and post‐mortem scanning electron microscopy (SEM) characterization [28]. During charging, the current tends to focus on surface heterogeneities leading to heterogenous Li deposition. Induced short‐circuits create a pathway for electrons through the electrolyte, but in most cases they are not permanent: a “thermo‐fuse effect” [36] allows cells recovery and further cycling. The importance of the Li electrode's microstructure was particularly revealed by an in situ SEM study of a bilayer pouch cell (positive/SPE/Li/SPE/positive (referred to as CELEC in the following text)) [35]. Hovington et al. highlighted the presence of electrolyte bridges with a too thin Li electrode: Li metal defects, such as grain boundaries, induce preferential Li stripping which creates holes in the Li electrode with isolated lithium island. Ion pathways, i.e. current density flows, impacted in part by Li metal heterogeneities and microstructure, therefore appear to be the key parameters in the end‐of‐life of ${\mathrm{LiFePO}}_{4}$/SPE/Li‐metal batteries.

To gain a better understanding of stripping and plating mechanisms in Li metal batteries using a SPE electrolyte, most of the studies focused on symmetric Li/SPE/Li (LEL) cells with only few correlation or discussion with the behaviour in complete batteries (LEC). However, Balsara's group revealed differences in cyclability and defects at the Li metal/SPE interface depending on the type of cells (symmetric LEL cells or batteries LEC) [28]. Different characterization techniques (electrochemical analyses or imaging analyses) are also developed. X‐ray computed tomography (XRCT), as a non‐intrusive 3D characterization technique, is a powerful tool to analyze the Li metal/SPE interface without damaging it. Balsara's group has published numerous studies characterizing symmetric cells using synchrotron XRCT [23, 31, 32, 37]. High‐quality images provide highly accurate measurements, while fast acquisition speeds facilitate in situ or operando characterizations. In a previous article, we demonstrated that post‐mortem studies of the Li/metal interface, using laboratory tomograph (more easily accessible to the scientific community), provide new information on stripping and plating heterogeneities [38]. A methodology was also described to obtain a quantitative characterization of the evolution of the Li metal electrode morphology after cell polarization.

XRCT is also reported in the literature to study the ${\mathrm{LiFePO}}_{4}$ positive electrode. This 3D analysis offers valuable information such as electrode microstructure (porosity) [39], or battery failure [40]. Being non destructive, the same sample can be analyzed at different scales. Carter et al. conducted a multi‐scale study on commercial ${\mathrm{LiFePO}}_{4}$ batteries using laboratory XRCT [41]. The different scales of analysis (from 9.77 µm to 218 nm of voxel size) allowed these authors to access to different types of information: macroscale defects, characterization of the composite electrode and detection of damage on the current collector, and finally detection of small variations of the electrode microstructure or interaction between the active material and the current collector.

Based on our previous methodology [38], the aim of the present study is to compare the end‐of‐life after cycling of SPE‐based Li symmetric cells (Li/Electrolyte/Li, i.e., LEL), with battery cells (Li/Electrolyte/Cathode, i.e., LEC; with the cathode being a ${\mathrm{LiFePO}}_{4}$ composite positive electrode) and with a more realistic bilayer cell (Cathode‐Electrolyte‐Li‐Electrolyte‐Cathode, i.e., CELEC) cells. The cells were assembled in pouch configuration (10 – 15 ${\mathrm{cm}}^{2}$). These three cell configurations were cycled at 80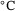 and 2 bar. A characterization after different lifetimes allows us to identify a mechanism for defect formation. Our study demonstrates the relevance of combining electrochemical analysis with X‐ray laboratory tomography imaging as an efficient post‐mortem battery analysis tool and emphasize the importance of linking analyses of symmetric, battery, and bilayer cells.

## 2 Results and Discussion

### 2.1 Electrochemical Signature of Cycling‐Induced Cell Failure

**FIGURE 1:**
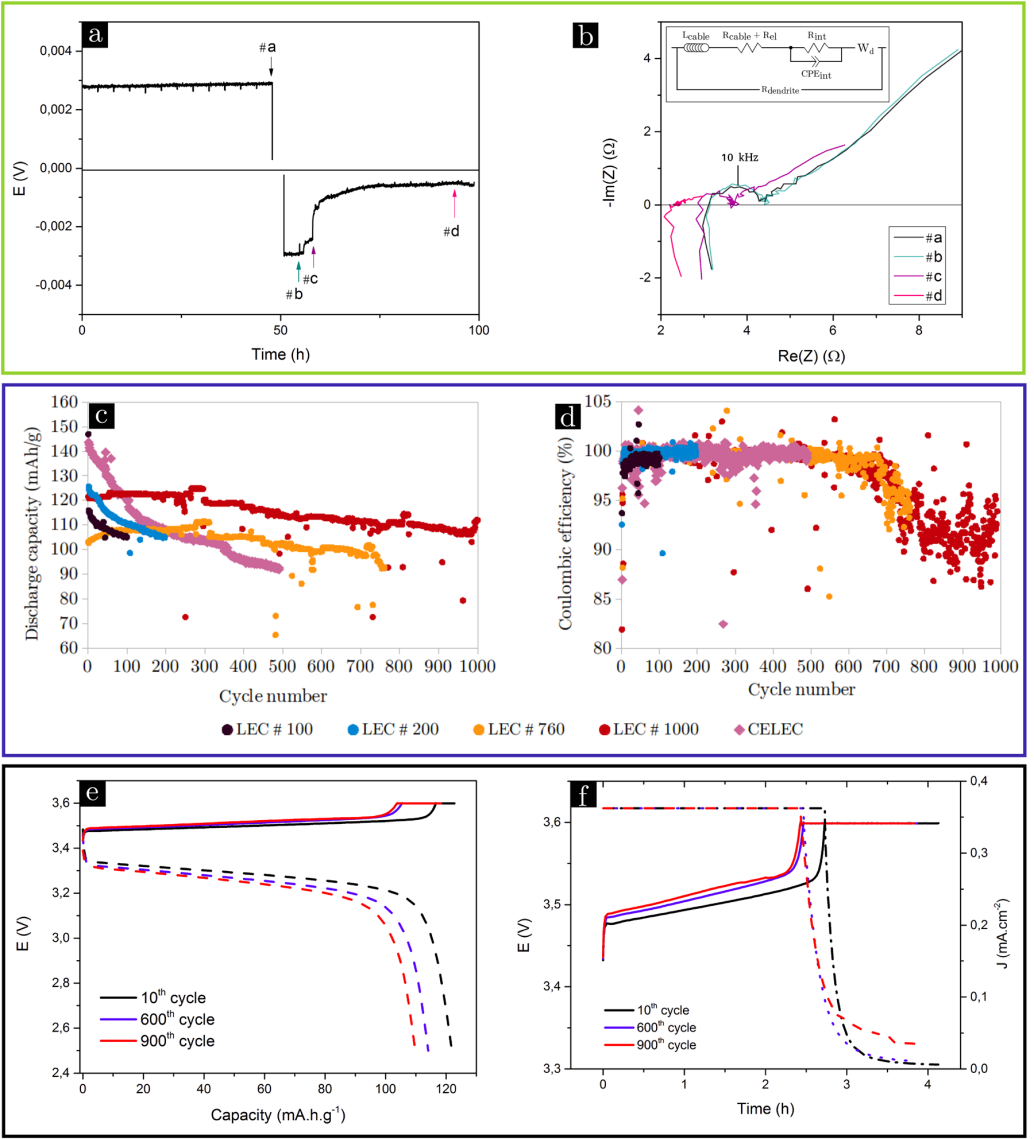
Difference in the electrochemical signature of LEL (green area) and LEC/CELEC (blue area) cell failures, and evolution of a LEC cycling performance (black area). (a,b) Electrochemical data of a prematurely short‐circuited LEL cell, polarized at a current density of 0.05 mA.cm^−2^ to displaced 12 µm of Li: (a) potential (E) monitored as a function of time during a complete cycle; (b) impedance spectra in Nyquist representation at different polarization times. The equivalent circuit for fitting these curves is the same as described in Figure S1 but modified to take into account the dendrite. Assuming that the short‐circuits are local, the macroscopic electrochemical properties are not impacted and the short‐circuit can be considered as a pure resistance ($R_{dendrite}$) in parallel, as proposed by Rosso et al. [36] (c,d) Comparison of the cycling data (cells cycled at C/4, D/2 rate) of the different LEC and CELEC stopped after different cycle numbers: (c) discharge capacity, (d) coulombic efficiency. (e,f) Electrochemical data of a LEC cycled for 1000 cycles at C/4, D/2 rate: (e) evolution of the cell potential with the capacity during the charge (solid line) and the discharge (dashed line); (f) evolution of the potential (solid line) and the current density (dashed line) with time during the charge.

LEL cells were assembled with a very thin SPE (about 14 µm, see scheme in Figure 6 in the experimental procedures section) which is realistic for a Gen4 battery, but can exalt any cross‐talking effects between electrodes [38]. Figure 1a shows the end‐of‐life of a typical LEL cell during galvanostatic cycling at a J value of 0.05 mA.cm^−2^. The LEL cell depicted in Figure 1a,b cycled 1/2 cycle without short‐circuiting and a short‐circuit occurred during the second reverse half‐cycle. Electrochemical Impedance Spectroscopy (EIS) spectra, recorded at different time positions (#a‐d in Figure 1a) are shown in Figure 1b. A short‐circuit is located at time position #c, associated with a sudden drop in the cell polarization. Prior the short‐circuit, the EIS spectra are all similar in shape and can be decomposed into high frequencies (HF, corresponding to cables and bulk of the electrolyte), medium frequencies (MF, characterizing phenomena at Li/SPE interfaces) and low frequencies (LF, providing information on ionic diffusion processes in the SPE [42].) Figure S1 displays the EIS spectrum of the initial state of a LEL cell with the electrical equivalent circuit used to fit the spectrum (see experimental part for details). The short‐circuit corresponds to complete changes of the EIS spectra recorded at position #c compared to the previous ones (#a and #b). Indeed, all the contributions $R_{el}$ (HF), $R_{int}$ (MF) and $R_{d}$ (LF) are reduced, but the reduction is more pronounced for LF processes. In #d, the short is more pronounced. The polarization being reduced to a fifth of the initial polarization (#a, #b), it is clear that the electrolyte resistance shifts to a lower resistance, and the total impedance is almost the fifth of the initial cell impedance (see Figure 1b). All the investigated LEL presents a similar failure due to a short‐circuit. Interestingly, all the current densities are lower than the limiting current density ($J^{*}$) beyond which dendrite growth is favored in presence of a solid polymer electrolyte [43].

**FIGURE 2:**
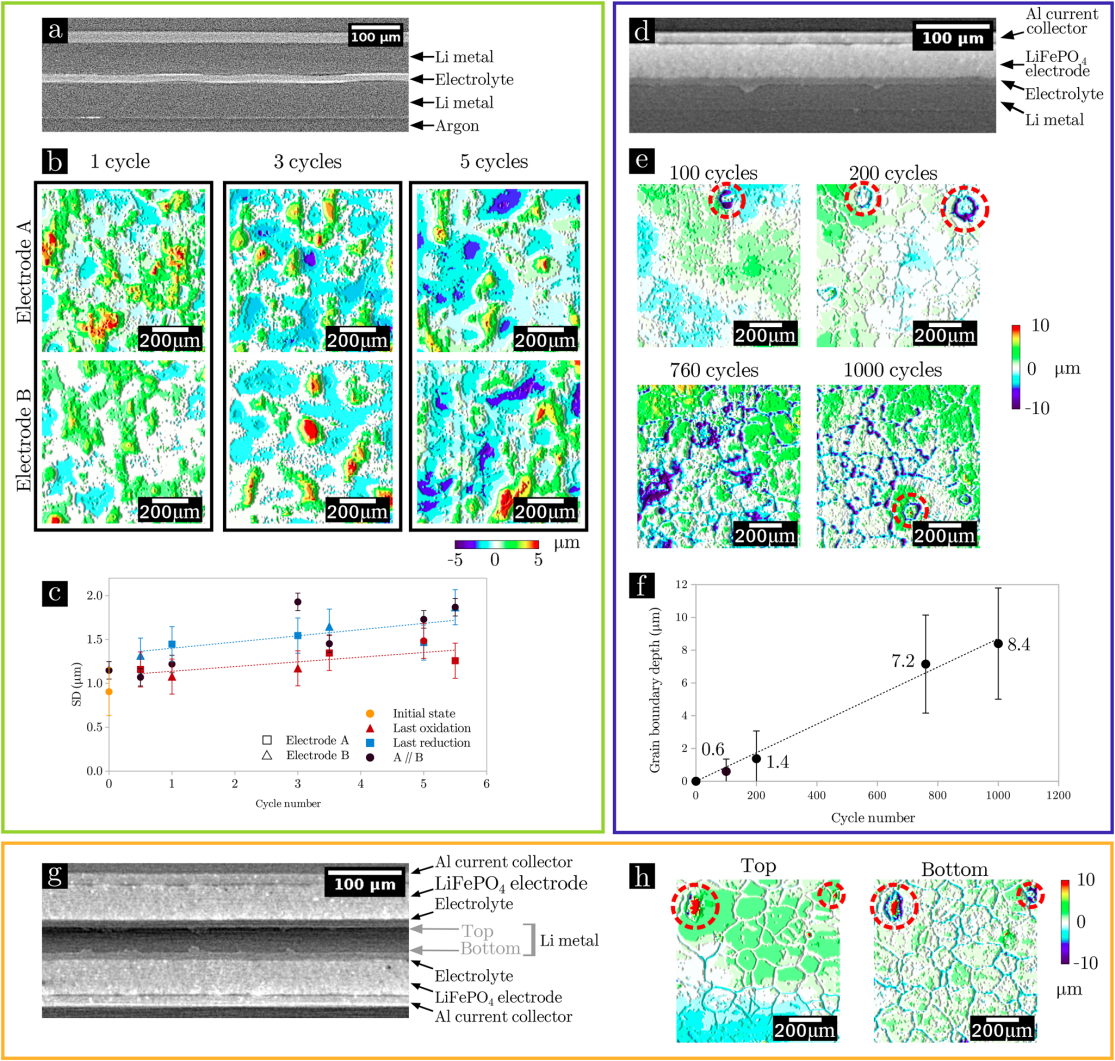
Li metal electrode thickness variations after cycling in LEL (green area), LEC (blue area) and CELEC (orange area) cells. (a–c) Study of LEL cells cycled at 0.05 mA.cm^−2^ to displace 12 µm of Li for different number of cycles: (a) (x,z) slices of XRCT image of the LEL cell cycled over three cycles; (b) 2D map (x,y) of Li thickness variations depending on the cycle number (these 900 × 900 µm 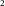 maps are a sub‐region of the total analyzed area, see Figures S3 and S4 for the other cycle numbers); (c) Influence of cycling on the standard deviations (*SD*) of thickness variations (extracted from the histograms plotted in Figure S5). (d–f) Study of LEC cells cycled at C/4, D/2 during different cycle numbers: (d) (x,z) slices of XRCT image of LEC#760 cell; (e) 2D map (x,y) of Li thickness variations depending on the cycle number (these 2D maps are a sub‐region of 1000 × 1000 µm 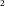 of the total analyzed area); (f) Evolution of grain boundary depth as a function of the cycle number. These quantifications correspond to the average of 20 measurements (difference between the thickness of a grain boundary and that of neighboring grains). A linear fit is given by the dash line. (g,h) Study of a CELEC cell cycled at C/4, D/2 during 500 cycles: (g) (x,z) slices of XRCT image of the CELEC cell; (h) 2D map (x,y) of Li thickness variations of the top and bottom surfaces of the Li electrode in the CELEC cell (these 2D maps are a sub‐region of 1000 × 1000 µm 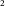 of the total analyzed area). In the 2D maps of thickness variations, the average thickness appears in white, higher thicknesses in green‐red and lower thicknesses in blue‐purple. Areas affected by inclusions are surrounded by a dotted red circle.

**FIGURE 3:**
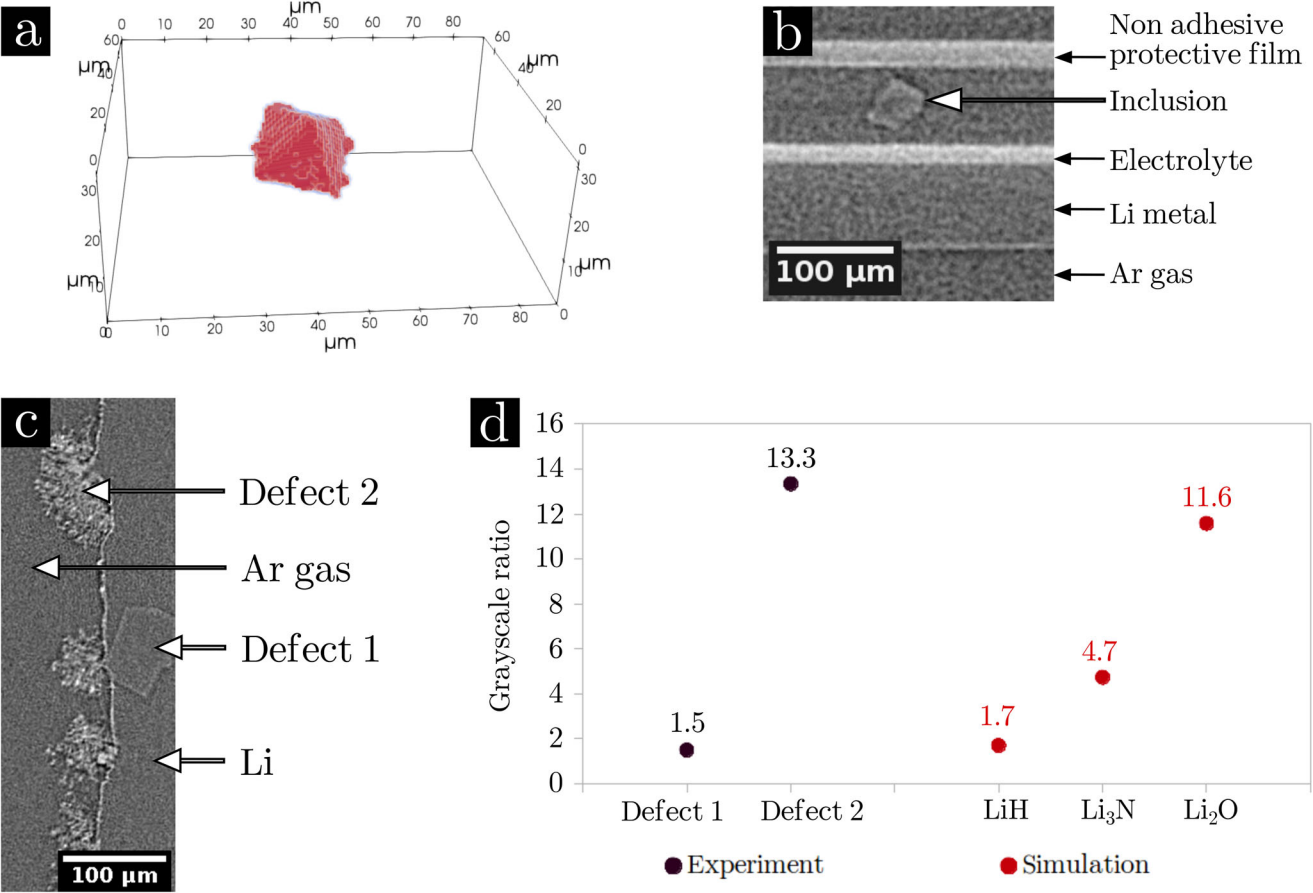
Characterization by XRCT of inclusions present in Li metal. (a) 3D rendering of the segmentation of an inclusion demonstrating its octahedral shape. (b) (x,z) slices of XRCT image of a LEL cell allowing comparison of the grey level of the inclusion with that of the electrolyte and the Li metal matrix. (c) XRCT slice of a Li metal foil submitted to slight surface oxidation containing two defects. (d) grey ratio calculated from Equation (2): experimental grey level ratio for the two defects present in the Li compared to the ratios obtained from the simulation at 20 keV (simulated grey values reported in Table S4).

**FIGURE 4:**
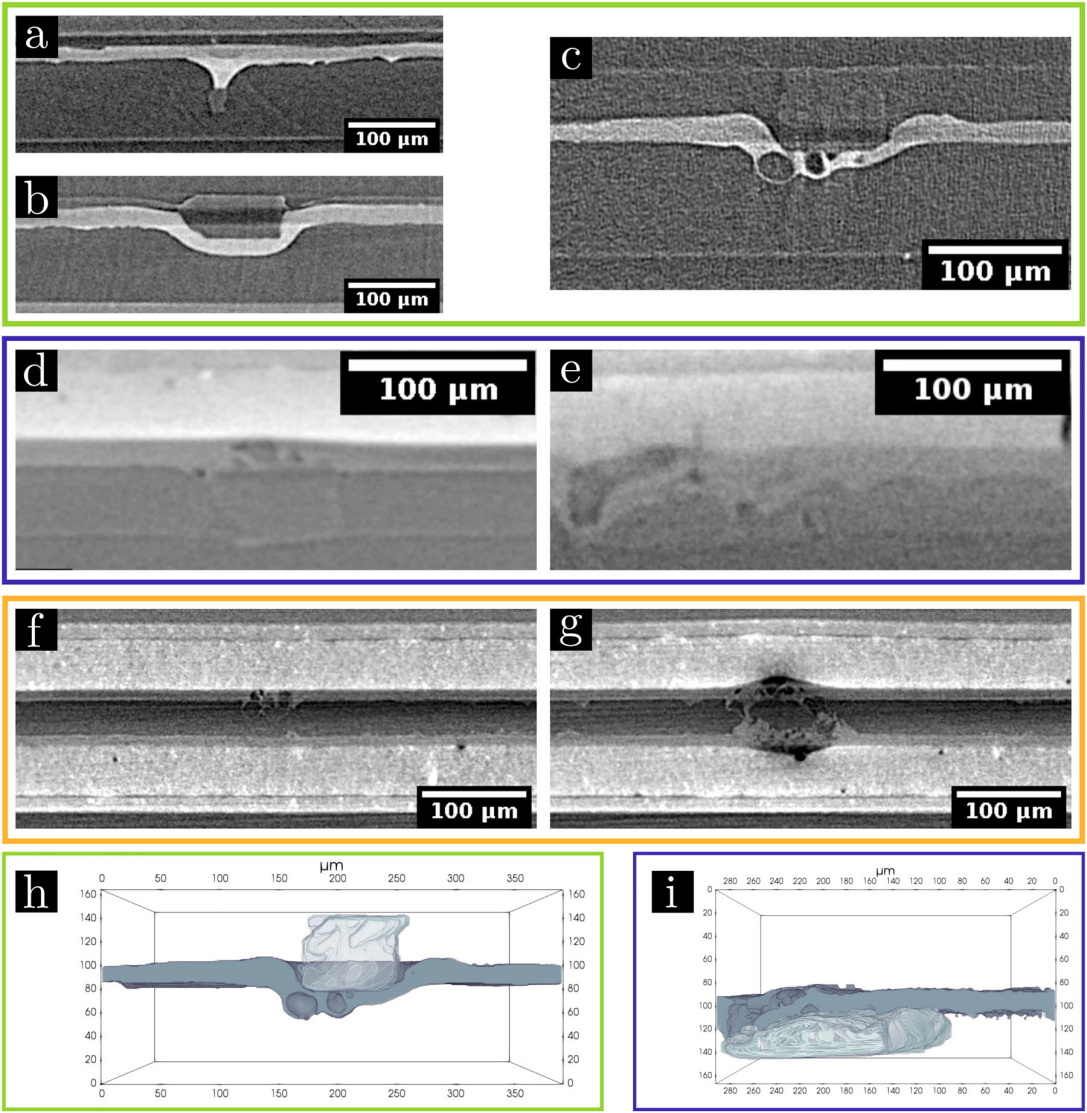
Defects created by inclusions (contained in Li metal) during cycling of LEL (green area), LEC (blue area) and CELEC (orange area) cells, characterized by XRCT. LEL cell polarized to move 40 μm of Li at 0.1 mA.cm^−2^ (Li displaced from the anode (top electrode) to the cathode (bottom electrode)) with the presence of inclusions in the electrodes: (a) in the cathode, (b) in the anode. (c) Characterization of the presence of globules at the base of an inclusion on a short‐circuited cycled cell after ten cycles (cycled at 0.05 mA.cm^−2^ with a displacement of 12 µm). LEC cells cycled at C/4, D/2 for: (d) 100 cycles and (e) 1000 cycles. CELEC cell cycled for 500 cycles with: (f) a small inclusion and (g) a larger inclusion. 3D rendering of the segmentation of the electrolyte (dark grey) and the inclusion (transparent light grey) in (h) a LEL cell (from Figure 4c) and (i) a LEC cell (from Figure 4e).

**FIGURE 5:**
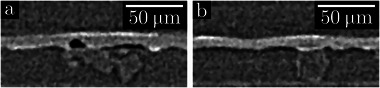
Illustration of the impact of inclusion size on defects created in a short‐circuited symmetric cell, cycled for three cycles at 0.05 mA.cm^−2^ with a displacement of 6.2 µm: (a) case of a large inclusion, (b) case of a small inclusion. Theses slices are extracted from the same symmetric cell, so Li electrodes that have undergone the same polarisation. A small inclusion does not modify the Li/electrolyte interface, whereas a large inclusion causes the formation of a globule.

**FIGURE 6:**
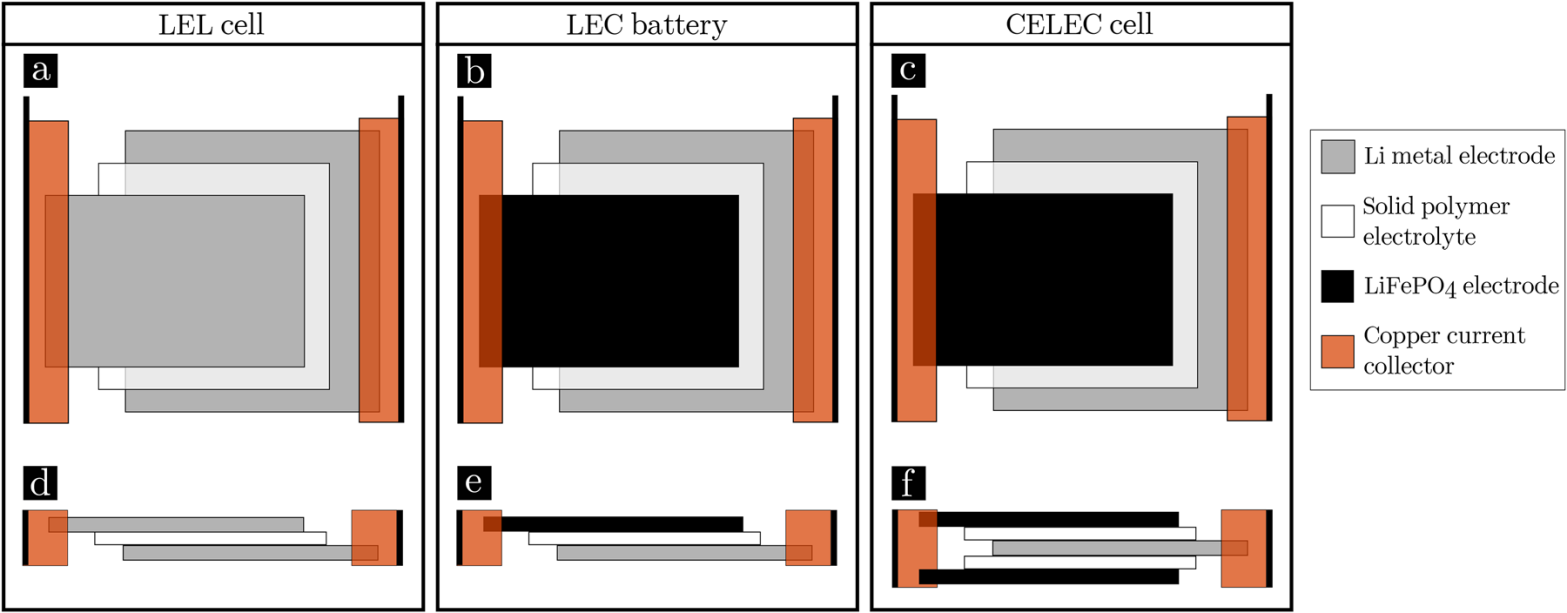
Cell assembly schematic: (a–c) top view, (d–f) cross‐section. Representations of the different type of cells: (a,d) symmetric LEL, (b,e) battery LEC and (c,f) bilayer CELEC cells.

Chazalviel proposed a model to describe the time before short‐circuit $t_{cc}$ for current density J higher than the limiting current density $J^{*}$ [44]. In this condition, the time before dendrite nucleation is the Sand time and is proportional to $1/J^{2}$. Chazalviel considers that the dendrite growth time is negligible compared to the nucleation time, so $t_{cc}$ should be similar to the Sand time. Since then, several studies demonstrate the dependency in $1/J^{2}$ of $t_{cc}$, for imposed current densities lower than $J^{*}$ [16, 36]. Due to heterogeneous interfaces (SEI, microstructure …), J is locally higher than $J^{*}$ which leads to local Sand conditions. The limiting current density, $J^{*}$, above which dendrite growth takes place is:

(1) $$J^{*}=\frac{2nFC_{0}D_{amb}}{(1-t^{+})L}\;\mathrm{with}\;t^{+}=\frac{\mu^{+}}{\mu^{+}+\mu^{-}}$$

with n = 1 the number of transferred electrons, F the Faraday's constant, $C_{0}$ the salt concentration, $D_{amb}$ the ambipolar diffusion coefficient, $t^{+}$ the cationic transport number (μ the mobilities) and L the inter‐electrode distance.

In our study, the limiting current density $J^{*}$ can be calculated from the analysis of impedance spectra where the low frequency region permits to extract the ambipolar diffusion coefficient ($D_{amb}$) (see experimental part for details). Based on several replicates, $D_{amb}$ is calculated to be $3.8\pm 0.1\times 10^{-8}$ ${\mathrm{cm}}^{2}$.$s^{-1}$; a value in agreement with a previous work (value of $5.8\times 10^{-8}$ ${\mathrm{cm}}^{2}$.$s^{-1}$) [45]. Moreover, the properties of the electrolyte are considered equivalent to a PEO homopolymer doped with LiTFSI at a salt concentration of EO:Li of 25, which gives a salt concentration $C_{0}$ of 882 mol.$m^{-3}$ [46]. The transference number ($t^{+}$) can also be assumed to be similar to PEO/LiTFSI and equals to 0.15 [46, 47]. Considering a 14 µm thick electrolyte, the limiting current density $J^{*}$ of the studied system is 5.5±0.2 mA.cm^−2^ based on Equation (1). This value is thus much higher than the experimental J used to cycle the LEL cells.

Comparatively, Figure 1c,d plot the cycling data (discharge capacity and coulombic efficiency) of different LEC cells cycled at a C/4 charge rate and D/2 discharge rate (see experimental section for details ‐ assembly scheme in Figure 6 and Table S1 for the list of the cells). The different cells were stopped after a fixed number of cycles to be analyzed post‐mortem by XRCT; this number is reported in the name of the cell (e.g., LEC#100 performed 100 cycles). As shown in Figure 1c, the capacity comparison highlights two types of evolution. First, a nearly constant capacity fade over the entire cycling (LEC#760 and LEC#1000); second, a significant drop in capacity during the first 50 cycles before reaching a more stable regime (LEC#100 and LEC#200). A capacity decrease is reported in literature in the case of a Li metal battery composed of a polymer electrolyte and a ${\mathrm{LiFePO}}_{4}$ positive electrode [28, 35]. This decrease is attributed to side‐reactions, causing active material degradation, formation of insulating materials and loss of contact leading to ${\mathrm{LiFePO}}_{4}$ particles becoming inactive. Although the capacities are distributed, LEC cell cyclability is good, with an average of 0.06 mAh.cm^−2^ of capacity loss per cycle. LEC#1000 loses 11.8 mAh.cm^−2^ of capacity over the 1000 cycles without any particular incident.

The coulombic efficiency, reported in Figure 1d, is stable upon cycling with a value of 99.7 ± 0.4 %. For both LEC#760 and LEC#1000, a drift of the coulombic efficiency toward lower values is observed after 600–650 cycles, without signature on the capacity monitoring curve (Figure 1c). At the end of cycling, the coulombic efficiency drops after 600 cycles around 5 % for LEC#760 (from 99.1 to 94.4 %) and 7 % for LEC#1000 (from 99.3 to 92.2 %). Herein, battery failure is characterized in each case by a drop in coulombic efficiency. The same observation was reported in the literature with a similar system (${\mathrm{LiFePO}}_{4}$‐based electrode and a polymer electrolyte): battery failure can be detected by a decrease in coulombic efficiency, but also by an abnormal drop in potential during the charge [28]. This latter failure was attributed to the presence of short‐circuits [28, 37].

Figure 1e,f focus on the charge/discharge curves before and after the drop in coulombic efficiency of the LEC#1000. As reported in Figure 1e, a small capacity loss is recorded with a slight increase in polarization over the first 1000 cycles. Figure 1f evidences: (i) the presence of local degradation of the cell operation [28] after 600 cycles (the current density decreases more slowly down to zero compared to the 10^*th*^ cycle); (ii) electronic leakages due to a soft short‐circuits, as reported by Hallinan et al. [28] for the 900^*th*^ cycle, i.e., after the drop in coulombic efficiency a residual current density ($j_{r}$) of 0.035 mA.cm^−2^ remains in the constant voltage step. Another evidence of the presence of short‐circuits is provided by Figure S2, representing the capacity variation (dQ/dE) as a function of the potential. For the 10^*th*^ and 600^*th*^ cycles, the capacity is mainly loaded onto the plateau at about 3,5 V (Figure 1e,f) with a broad peak in dQ/dE in between 3,48 et 3,54 V (Figure S2). On the other hand, for the 900^*th*^ cycle, small steps can be seen at the ends of the load (Figure 1f), giving a new contribution of around 3.53 – 3.54 V on the dQ/dE versus E graph (Figure S2). All these features, $j_{r}$ and bumps easily detectable in dQ/dE, reflect the presence of soft short‐circuits. Thus, direct electronic transfer between the Li electrode and the ${\mathrm{LiFePO}}_{4}$ electrode seems to occur after a certain period of maturation that would explain the drop in coulombic efficiency between the 600^*th*^ and 900^*th*^ cycles.

In electric vehicles application, Li metal electrodes are sandwiched between two positives electrodes (“cathodes”) so that each side of the Li foil can work electrochemically, maximising energy density. A laboratory scale study of the impact of this assembly called CELEC (see scheme in Figure 6c,f) is carried out using the same methodology. The performance of the CELEC cell (cycling stopped after 500 cycles) is reported on Figure 1e,f. Despite an abnormal drop in discharge capacity, the evolution of coulombic efficiency is identical over the first 500 cycles with LEC#760 and LEC#1000. Therefore, the bilayer assembly does not induce premature death of the battery (no soft short‐circuit).

These electrochemical data highlight a difference between LEL and LEC/CELEC cells cycling behavior: different phenomena are responsible for failure, depending on the cell type. LEL cells can only cycle for a few dozen cycles at most before a definitive short‐circuit occurs, whereas LEC and CELEC cells can cycle for several hundred cycles before a soft short‐circuit appears. Moreover, the current densities suitable for cycling are very different depending on the type of assembly: LEL cells must be cycled at low current densities (0.05 mA.cm^−2^) to achieve several cycles, while LEC cells can be cycled at C/4 (0.36 mA.cm^−2^), D/2 (0.73 mA.cm^−2^). This higher cyclability of batteries compared to symmetric cells is in agreement with the literature, which ascribed this phenomenon to a better current homogeneity in LEC cells [28] or the effect of correlated roughening of both electrodes in the symmetric cells [38].

### 2.2 Influence of Grains and Grain Boundaries on Li Electrode Morphology Upon Cycling

As reported in Table S2, both a low J and a small exchanged capacity (low Li thickness displaced ($th_{1/2}$ 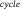)) are less likely to induce short‐circuit during LEL cycling. Knowing these cycling limitations, another study was carried out by assembling new LEL cells (see Table S3) that were cycled at ± 0.05 mA.cm^−2^ with 12 µm of Li exchanged at each half cycle. Each of the seven cells was polarized for a different cycle number (0, 1/2, 1, 3, 3+1/2, 5 and 5+1/2 cycles) without short‐circuit, and further analyzed by XRCT to investigate the impact of the Li stripping and plating cycles on the morphology of the Li metal/SPE interface. This interface is well defined in XRCT scan (Figure 2a) and 2D maps of electrode thickness variations can be calculated (Figure 2b; Figures S3 and S4). We measured the uncertainties associated with image analysis by performing a sensitivity analysis. According to Figure S6, the variation in the threshold value induces an uncertainty of one voxel, i.e., 1 µm, on the position of the interface. This uncertainty then applies to all subsequent morphological parameters calculated from XRCT image analysis. The electrode named electrode A is the one whose first redox process is oxidation, and conversely, the electrode B is the one subjected to reduction first. At the end of a half cycle (“charge”), the last process taking place at the electrode A (respectively electrode B) is oxidation (respectively reduction). At the end of a complete cycle (“discharge”), the last process at the electrode A (respectively electrode B) is reduction (respectively oxidation). The image analysis methodology, described more precisely in our previous publication [38], consists in the selection of the Li/SPE and Li/argon interfaces. The distance between these two interfaces is calculated to measure the Li electrode thickness in each point of the analyzed volume. This value can thus be represented as a 2D map showing the local variations around the mean thickness of the Li electrodes. This 2D map gives qualitative information on local heterogeneities induced by cell cycling. The distribution of the Li electrode thickness on the 2D map can be plotted (see Figure S5). The standard deviation of this distribution is chosen as a quantitative value to compare the heterogeneities of different 2D maps (Figure 2c): a higher standard deviation (*SD*) evidences higher heterogeneities. The uncertainties on the SD values, equal to 0.2 µm, were estimated by considering the impact of the threshold on the variation quantification, the repeatability of the measurements from one cell to another, and the representativeness of the analyzed area (see details in Figures S6–S9).

In the initial state, very small thickness variations are randomly distributed over the electrode surface (see Figure S3) with a relatively small *SD*. The value reported in Figure 2.c is 0.9 ± 0.3 µm corresponding to the average of the two electrodes at the initial state and the associated standard deviation. After 1/2 cycle (see 2D maps in Figure S4), few variations of the topography (5 µm higher than the reference level) appear, with large valley 3 µm below the reference level. Their shape and configuration are correlated to the Li microstructure, i.e., some grains are in average thicker than others (this correspondence was thoroughly demonstrated in our previous publication [38]). Cycling increases heterogeneities with deeper valleys (‐ 6 µm) and mounts (+ 6 µm), which translates in an increase in SD with the number of cycles. These variations are considerable, accounting for 50 % of the thickness displaced each half‐cycle. Reduction in the last process leads to more heterogeneities than oxidation. Oxidation in the last process tends to smooth the surface heterogeneities induced by the reduction in the previous half cycle. After five cycles, both electrodes have the same order of magnitude of thickness variations (similar *SD*). In our previous publication [38]. we demonstrated that the interdependence of heterogeneities on the two Li electrodes was highlighted by the calculation of the A//B mapping (subtraction of the local thickness variation of the electrode A by that of the electrode B in each point of the 2D map). The presence of cross‐talking is highlighted by comparing the 2D map histograms: the two electrodes are interdependent (cross‐talking) if *SD* 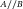 is lower than *SD* 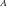 and *SD* 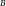. On Figure 2.c, the values of *SD* 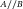 indicate that cross‐talking is present during the half cycles (1/2, 3+1/2 and 5+1/2 cycles): *SD* 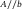 is lower than that of the electrodes A and B taken separately. Cross‐talking is less present at the end of full cycles (1, 3 and 5 cycles) and it does not seem to be impacted by the increase in the number of cycles. These results suggest that the plating/stripping local kinetics is impacted by cross‐talking between the two Li electrodes which is driven by the heterogeneities grains to grains such as grain orientation, SEI nature, and local stress [21, 23]. In addition, the plating systematically leads to higher heterogeneities than the stripping at this low rate. For comparison, the same approach is applied to the LEC cells in the next paragraph.

The four LEC cells (Table S1) cycled for a different cycle numbers from 100 to 1000 cycles were characterized post‐mortem by XRCT. The ${\mathrm{LiFePO}}_{4}$ positive electrode used here contains highly X‐ray‐absorbing materials. Despite this drawback for X‐ray transmission, Figure 2d proves that, with optimized acquisition parameters, the Li/SPE interface remains clearly visible even with a laboratory tomograph. The quality of the contrasts obtained in LEC (i.e., with the positive electrode highly absorbing) is however degraded compared to the very good resolutions obtained for symmetric LEL cells where all the materials have a low attenuation coefficient. The positive electrode attenuates much more and then causes shadowing effects. No sensitivity tests were thus performed in the LEC case. Note that consequently in this case, we also didn't perform any quantitative measurement from the 2D maps. We limited ourselves to manual measurement of grain boundaries depth from the 3D images. It is also interesting to note that the presence of the electrolyte, slightly more absorbent than Li, reduces the shadowing effect of the positive electrode, and the Li/electrolyte interface remains well defined and contrasted. 2D maps showing the evolution with the cycle number of the local thickness of the Li metal electrode in LEC cells are reported in Figure 2e. After 100 cycles, the Li electrode is globally smooth with an homogeneous thickness. The main defect (surrounded by a dotted red circle) is related to the presence of an inclusion that will be discussed later. After 200 cycles, some grain boundaries begin to become less thick creating valleys and the electrolyte continues to stick to these created grooves. While cycling, grain boundary depth become larger from 1.4 ± 1.6 µm after 200 cycles to 8.4 ± 3.4 µm after 1000 cycles. Interestingly, the width of the valleys is globally constant between 30 and 40 µm. The quantitative analysis in Figure 2f shows a trend toward a linear increase in grain boundary depth with the cycle number (within the error bars). After 1000 cycles, the grain boundaries are 8.4 µm deep on average (greater than the 6.2 µm displaced Li thickness every half cycle with the ${\mathrm{LiFePO}}_{4}$‐based positive electrode used in our study).

The same analysis was also performed for a more realistic bilayer CELEC cell, where the Li metal is cycled on both sides of the electrode as shown on the XRCT scan in Figure 2g. The electrochemical characterization (Figure 1c,f) displays a similar evolution as LEC cells (no coulombic efficiency drop during the 500 cycles performed). In Figure 2h, 2D maps of the evolution of the electrode morphology reveal that Li grain boundaries are also grooved on both sides of the Li electrode. Interestingly, very similar grain boundary pattern is visible on both sides of the Li electrode, which suggests that Li foil contains mostly columnar grains. The average grain boundary depth on each side shows a fair agreement with 3.5±1.9 µm and 3.7±1.4 µm for the top and bottom side, respectively. The small gap between the two values can be explained by a small unbalance between the upper and lower cathodes that are branched in parallel so the imposed current is averaged over the two electrodes. The grains being columnar, this induces a symmetry in the valleys created. Herein, by combining the two valleys, we estimate that an average of 24% of the electrode thickness is dug at grain boundaries after 500 cycles.

### 2.3 Inclusion‐Induced Defects at Li/SPE Interface

The presence of inclusions in Li metal (octahedral in shape see Figure 3.a; from ten to hundreds of micrometers, see Figure S10) was already shown by Harry et al. [23] Their chemical composition remains uncertain and discussed in the literature as containing hydrogen, oxygen and/or nitrogen. The following compositions are proposed: ${\mathrm{Li}}_{2}O$, LiOH [23] or LiH [48, 49]. Figure 3b shows that the grey level of the impurity is very close to that of the Li metal matrix, in comparison to the solid polymer electrolyte PEO‐LiTFSI 1M (mainly composed of light elements), which appears in very clear contrast. This means that the impurity is much less absorbent than the electrolyte and similar to lithium. Figure 3c displays an XRCT image containing Li metal, an inclusion (defect 1) and an object formed by surface oxidation (defect 2). The XRCT image contrasts can be used to determine the chemical composition of the inclusion. Using the GATE software [50], the X‐ray attenuation coefficients μ of the compounds LiH, ${\mathrm{Li}}_{3}N$ and ${\mathrm{Li}}_{2}O$ were simulated to estimate their grey values, calculated by a Monte Carlo simulation (reported in Table S4). The following ratio ($R_{inclusion}$) can be calculated, with G the grey level of the different compounds relative to the one of Argon (Ar):

(2) $$R_{inclusion}=\frac{G_{inclusion}-G_{Ar}}{G_{Li}-G_{Ar}}$$

These ratios, calculated from simulations of the LiH, ${\mathrm{Li}}_{3}N$ and ${\mathrm{Li}}_{2}O$ attenuation, are plotted in Figure 3b as “simulation” data. $R_{inclusion}$ was also calculated using the experimental grey value (measured from Figure 3a) and are reported as “experimental” data in Figure 3d. The comparison of the experimental ratios to those simulated indicates that defect 1 appears to be LiH while defect 2 would be ${\mathrm{Li}}_{2}O$ (or a heavier compound such as ${\mathrm{Li}}_{2}\mathrm{CO}$ 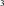). The slight discrepancy between experiment and theory is due to the polychromatic effect of the beam. This simulation is one strong argument in attributing the LiH nature to the inclusions.

The presence of inclusions causes very important heterogeneities at the Li/SPE interface after Li polarization in LEL cell (see Figure 4a,b). As LiH [51], these inclusions are insulating, as reported in the literature [23, 48]. When emerging at the interface Li/SPE, they block the Li plating on their surface leading to the formation of a hole in the plated Li with a trumpet shape filled by the electrolyte (Figure 4a). During stripping in front of the inclusion, the cathode presents a replicate of the footprint of the insulating inclusion, as the inclusion is not oxidized (Figure 4b). In both situations, the electrolyte is strongly distorted to accommodate these interfacial heterogeneities around the emerging inclusion upon Li plating or stripping. With cycling, another type of defect is observed at the Li/SPE interface as shown in Figure 4c,h: globules grow at the base of inclusions within the electrolyte leading to a short‐circuited LEL cell [37]. This suggests that the chemo‐mechanical stress of the SPE at the edge of the inclusion has a huge impact, as it is repeated at each cycles. It induces the formation of globules formed by ovoid balls of lithium (grey) at the edge of inclusion and/or formed by gas (dark) at the tip of inclusion, both surrounded by a polymer layer. In good agreement with previous work [23] these globules around the inclusion are the origin of the quick short‐circuit between the two Li metal electrodes.

In LEC cells, inclusions induce defects at the Li/SPE interface for all characterized lifetimes (100, 200, 760, and 1000 cycles, see Figure 4d,e; Figure S11). The size and the volume of the globule are larger after a higher cycle number. As in LEL cells, a globule is a complex microstructure of Li metal and electrolyte components in the Li electrode and in the electrolyte (Figure 4e). A given globule can grow with cycling to finally be in contact with the ${\mathrm{LiFePO}}_{4}$ electrode (as visible in Figure 4e,i).

Figure 4f illustrates the defect induced by the presence of a small inclusion at the Li/SPE interface in CELEC cell. As for the LEC study, the presence of an inclusion in the Li electrode locally perturbs the Li/SPE interface. The induced defect depends on the inclusion size. Indeed, if the inclusion is large enough, it can be in contact with the electrolyte on both surfaces of the electrode. The induced defects are therefore present on both surfaces of the Li electrode as shown in Figure 4g.

### 2.4 Different Failure Mechanisms for the Various Cell Types

Thickness variation maps in inclusion‐free zones (Figure 2) reveal a difference in Li metal behavior under stripping and plating depending on the cell type: (i) in LEL, heterogeneities are present from one grain to another or within the same grain; (ii) in LEC, thickness variations are observed between grain and grain boundaries. This can be explained by the type of electrode facing the working Li metal electrode. In LEL cells, the Li metal faces another Li metal electrode. Current densities are heterogeneous from grain to grain and cross‐talking between the electrodes demonstrates the presence of interaction between the two electrodes. Local variations in the impedance of Li metal electrodes (due to SEI, grain boundary …) are enhanced by this cross‐talking phenomenon and therefore amplify the created defects. This is explaining the induced thickness variations representing 50 % of the thickness displaced at each half‐cycle in Figure 2. In LEC cells, the ${\mathrm{LiFePO}}_{4}$ positive electrode induces currents that are likely to be more homogeneous (particle diameter 1 µm much smaller than Li grain size), so variations between grains and grain boundaries become preponderant compared to the differences between individual grains. For CELEC cells, valleys at grain boundaries can, in the long term, deepen over the entire thickness of the Li electrode, leading to a loss of electrical contact in the direction transverse to the thickness of the electrodes and then to a (minor but substantial) loss of battery capacity. Such observation was reported in literature: Hovington et al. have shown in CELEC the formation of an electrolyte bridge through the Li electrode, with isolated isles of Li if the film is too thin [35]. When the electrode is thicker, this phenomenon might be prevented by the increase of the electrolyte resistance at grain boundaries, which would limit the grain boundary depth. To date, few studies have focused on the differences in Li metal behavior depending on the assembly type. However, to extend battery lifetime by understanding Li metal behavior upon cycling, we demonstrate that the right type of cell must be selected depending on the scientific investigation.

Inclusions in the Li metal electrode lead to globule growth in each of the three cell types studied (Figure 4). Globule growth can be identified as the cause of short‐circuit in LEL cells as reported in the literature [23, 31]. Figure 4a,b illustrates that, being insulating, the inclusions induce modified local current densities and high mechanical stresses on the electrolyte. The composition of this mixture of Li and electrolyte is in agreement with the impedance data in Figure 1b. The impedance fits at points #c and #d give a relatively high short‐circuit resistance $R_{dendrite}$ = 20 Ω and $R_{dendrite}$ = 4.9 Ω, respectively. This suggests a very thin dendrite or a poor contact (low conductivity of the globule). In our study, the cell polarization is in the low current density range ($J/J^{*}=6\times 10^{-3}$). When moving Li in only one direction, no globule was observed on any of the cells studied (Figure 4a,b), which is in agreement with the observations of Maslyn et al. (no globule growth when $J/J^{*}<0.02$) [37]. Globules were observed only in the case of cycled cells (Figure 4c): their formation is therefore caused by repeated stripping and plating, as described by the group of Balsara [23, 31]. Figure 5a represents the cycling of a LEL cell, where the Li capacity moved per half‐cycle (corresponding to a Li displacement of 6.2 µm) is the same as in LEC cycling. The presence of globules after three cycles proves that globules grow faster in LEL than in LEC cells. An interesting result is observed in Figure 5: the larger the inclusion, the faster the growth. Indeed, in the same cell, no globule is observed with a small inclusion, whereas globule growth occurs with a larger inclusion. A correlation between the inclusion size and the degradation speed seems to exist. The position of the inclusions relative to the Li/electrolyte interface is also crucial. Maslyn et al. demonstrated that removing the inclusions at the Li/electrolyte interface drastically improves the cycling time of LEL cells [33]. Further studies need to be conducted by controlling the size of the inclusions and their position relative to the Li/electrolyte interface in order to determine a statistical law on the impact of inclusion size and volume fraction on battery cycling.

LEC cells failure is also directly related to the presence of inclusions in Li: local current densities are disturbed and this leads to globule growth and induces short‐circuit. Impedance measurements (Figure S12) demonstrate that the ${\mathrm{LiFePO}}_{4}$ positive electrode does not appear to be impacted by cycling. Compared to LEL cells, a slower globule growth and thus an increased time before short‐circuit may be induced in LEC cells by: (i) the absence of cross‐talking, (ii) the more homogeneous current densities (due to the higher uniformity of the ${\mathrm{LiFePO}}_{4}$ electrode facing the metallic Li foil) and (iii) the more rigid positive electrode (minimizing the mechanical deformation of the electrolyte). As LEC cells, CELEC cells failure can be attributed to globule growth, but cycling on both sides of the Li electrode can induce defects at both Li/SPE interfaces depending on the size of the inclusions (Figure 4f,g), leading to further capacity loss. The extreme current densities passing through a very localized electrical contact can create a fuse effect, explaining the soft character of short circuits. We can hypothesize that the absence of this phenomenon in LEL cells is due to the faster kinetics of globule growth: their extremely rapid formation creates a faster and stronger electrical contact, leading to an abrupt short‐circuit of the LEL cell.

## 3 Conclusion

Symmetric (LEL) cells are often studied in the literature to characterize Li metal behaviour as a way to improve battery performance. This assembly has the advantage of rapid electrochemical test (days or weeks), and good X‐ray contrasts in X‐ray computed tomography (XRCT) images. On the contrary, batteries (LEC cells) cycling is longer (several months) and XRCT contrasts are degraded by the presence of heavy elements in the positive electrode. The present work compares these different cell designs and demonstrates a difference in the electrochemical signature of cell end‐of‐life and in Li metal morphology evolution in LEL and LEC cells. On the one hand, LEL cycling induces heterogeneities from one grain to another, which increase with the number of cycles and are more important after reduction (compared to oxidation). On the other hand, during LEC cycling valleys are progressively grooved at the grain boundaries, which depth increases with the number of cycles. LEL can cycle a limited number of cycles (less than ten cycles) before an abrupt short‐circuit. LEL cell XRCT analysis reveals the presence of insulating inclusions leading to globule growth responsible for these short‐circuits. LEC cycling can be performed on a much higher number of cycles (several hundred of cycles) before the appearance of a soft short‐circuit. Slow globule growth at the Li metal/polymer interface is identified as the cause of battery failure due to the presence of insulating inclusions in the Li metal. The comparison of the experimental grey levels of the XRCT images with simulated values of possible grey levels reveals that these inclusions are probably LiH. A test representative of a real cell in application using a Li metal electrode sandwiched between two positive electrodes (bilayer CELEC cell) was also investigated and demonstrated the same type of defects as in LEC. The importance of grooves at grain boundaries is even more critical in bilayer cells, as the columnar shape of Li grains can eventually induce isolated Li grains. These different results underline the importance of analyzing battery or bilayer assembly to understand failure in conditions close to the actual application. This study also highlights the importance of controlling and limiting the inclusions presence or size in Li metal, responsible for cell failure.

## 4 Experimental Section

### 4.1 Cell Assembly

#### 4.1.1 Materials

Li electrodes were supplied by Blue Solutions company with a thickness of 30 or 60 µm, with an accuracy of ± 5 µm. The electrolyte used was a poly(ethylene oxide)‐based solid electrolyte layer (around 14 µm thick) also supplied by Blue Solutions. The positive electrode, formulated by Blue Solutions, was a ${\mathrm{LiFePO}}_{4}$‐based composite, providing a capacity of 150 mA.h.$g^{-1}$.

#### 4.1.2 Li Symmetric Cell (LEL) Assembly

Li symmetric cells comprising a solid polymer electrolyte were assembled in a dry room. There, a Li metal foil (60 ± 5 µm thick) was pressed onto the electrolyte and then laminated to ensure good adhesion. At each lamination step, the Li surface in contact with the laminating roll was protected using a non‐adhesive film. A second Li foil was added onto the electrolyte side before a second laminating step. Copper current collectors were then attached on both Li electrode edges to be separated from the electrochemical active surface that will be later on cut for X‐ray imaging purpose. The resulting assembly is shown in Figure 6a,d. After assembly, the Li symmetric cell was placed in a pouch bag and vacuum‐sealed.

#### 4.1.3 Battery (LEC) Assembly

Batteries (Li‐Electrolyte‐Cathode, LEC) were assembled using the same protocol as the Li symmetric cells. The difference was that one electrode was made of a ${\mathrm{LiFePO}}_{4}$ based composite positive electrode (referred to as the cathode), as schematized in Figure 6b,e. The Li metal foil was 30 ± 5 µm thick. This thickness differs from that used for LEL cell study. However, this difference did not affect the results because: (1) the phenomena observed were occurring at the interface (variations in thickness over the first few µm of Li thickness), and (2) other LEL cells had been cycled with thicknesses of 30 µm and demonstrate the same characteristics.

#### 4.1.4 Bilayer Battery (CELEC) Assembly

The bilayer cell was composed of a cathode‐electrolyte‐Li‐electrolyte‐cathode (CELEC) stack. Two samples of the ${\mathrm{LiFePO}}_{4}$ electrodes were cut with the same dimensions. A first cathode layer was pressed onto the electrolyte and rolled. The Li foil (30 ± 5 µm thick) was added onto the electrolyte before a second rolling step. A second electrolyte layer was pressed onto this Li foil before rolling. Finally, the second cathode layer was added onto the electrolyte for a last rolling step. The two cathodes were strictly superimposed and a top view scheme is presented in Figure 6c. Copper current collectors (foils) were attached to the Li foil on one side, and to both cathode layers on the other side (see Figure 6f).

### 4.2 Electrochemical Methods

#### 4.2.1 Cell Preparation

Before cycling, each cell (LEL, LEC and CELEC) was placed in a compression homemade cell holder applying a constant external pressure of 2 bar. It was then placed in an oven (Memmert) held at a temperature of 80 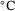, and the current collectors were connected to a VMP3 potentiostat (BioLogic). The cells were placed in the oven for 5 h before cycling, to ensure temperature homogenization.

#### 4.2.2 LEL Cycling

To characterize the initial state, the cell impedance was recorded via potentiostatic electrochemical impedance spectroscopy (PEIS). At the open circuit voltage, an excitation signal of 10 mV was applied to the cell in a frequency range in between 1 MHz and 3 mHz. Then, the LEL cells were galvanostatically polarized at a constant current density in order to move Li from one electrode (the anode) to the other (cathode). The applied current (I) was chosen according to the desired current density (J). The polarization time (t) was calculated based on the desired displaced thickness (th) using the Faraday equation:

(3) $$t=\frac{F\times \rho_{Li}}{J\times M_{Li}}\times th$$

with F the Faraday constant, $\rho_{Li}$ the Li density (0.534 g.${\mathrm{cm}}^{-3}$) and $M_{Li}$ the Li molar mass (6.94 g.${\mathrm{mol}}^{-1}$).

Then, during cycling, every 4 h, the cell impedance was measured by galvanostatic electrochemical impedance spectroscopy (GEIS). An oscillation of ± 20 % of the imposed current was applied in a frequency range in between 200 kHz and 50 mHz. Depending on the study, one or several cycles were performed. After total Li displacement in one direction, a PEIS was performed. To displace the Li in the opposite direction, the imposed current was inverted and GEIS measurements also carried out every 4 h. The final state was characterized by a last PEIS measurement at open circuit voltage.

#### 4.2.3 Impedance Interpretation

Figure S1 displays the impedance spectrum as a Nyquist representation of the initial state of a LEL cell. Quantitative measurements could be extracted from the fit of this curve. This fit could be obtained with the equivalent circuit drawn on the figure comprising: cable inductance ($L_{cable}$), cable and electrolyte resistance ($R_{cable+el}=R_{cable}+R_{el}$), interface resistance ($R_{int}$) in parallel with a constant phase element ($CPE_{int}$), and in series with a short Warburg ($R_{d}$, $\tau_{d}$). The electrolyte resistance ($R_{el}$) appeared at the highest frequencies (left side of the spectrum, around 105 Hz). It was disturbed by the presence of cables ($L_{cable}$, $R_{cable}$). It was therefore difficult in our systems to have a precise measurement of the electrolyte resistance unless the resistance of the cables was measured independently. The frequency at the top of the first loop (around 103 Hz) is typical of the presence of a passive layer and phenomena at the interfaces ($R_{int}$, $CPE_{int}$). Transport processes ($R_{d}$, $\tau_{d}$) were characterized by lower frequencies (right side of the spectrum, top frequency around $10^{-3}$ Hz). The frequencies were in agreement with data reported in literature [42]. In our study, the interest of such an analysis was the calculation of the diffusion coefficient ($D_{amb}$). It depends on the relaxation time ($\tau_{d}$) and the thickness of the electrolyte (L) [42, 52]:

(4) $$\tau_{d}=2.54\times \frac{{\left(L/2\right)}^{2}}{D_{amb}}$$

#### 4.2.4 LEC and CELEC Cycling

The battery practical capacity ($C_{p}$) is calculated using the following formula:

(5) $$C_{p}=C_{theo}\times S\times th_{c}\times w_{LFP}\times d$$

with $C_{theo}$ the ${\mathrm{LiFePO}}_{4}$ nominal capacity (150 mA.h.$g^{-1}$), S the active surface, $th_{c}$ the positive electrode (cathode) thickness, $w_{LFP}$ the weight percent of ${\mathrm{LiFePO}}_{4}$ active material, and d the ${\mathrm{LiFePO}}_{4}$ density.

The battery initial state was characterized by a PEIS at the open circuit voltage using an excitation signal of 10 mV in a frequency range in between 1 MHz and 50 mHz. Then, a pre‐cycling at 80 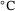 with a constant low current density was performed for five cycles at a C/10‐D/10 rate (charge‐rate “C” and discharge‐rate “D” in 10 h). A charge cut‐off voltage limit at 3.6 V was imposed to prevent electrolyte degradation followed by a holding step still at 3.6 V for 1.5 h to charge the battery at its maximum. A relaxation step of 30 min was then performed including a PEIS measurement in its middle prior to the discharge step. For the discharge, a cut‐off voltage limit at 2.5 V was imposed. No potential holding was maintained after discharge and a PEIS measurement characterized the end of discharge state (recorded during the subsequent relaxation 30 min step). After this pre‐cycling, the batteries were cycled with a C/4‐D/2 rate for several hundred cycles using the same protocol including the relaxation and PEIS steps.

### 4.3 X‐ray Tomography Imaging

#### 4.3.1 Sample Preparation

**FIGURE 7:**
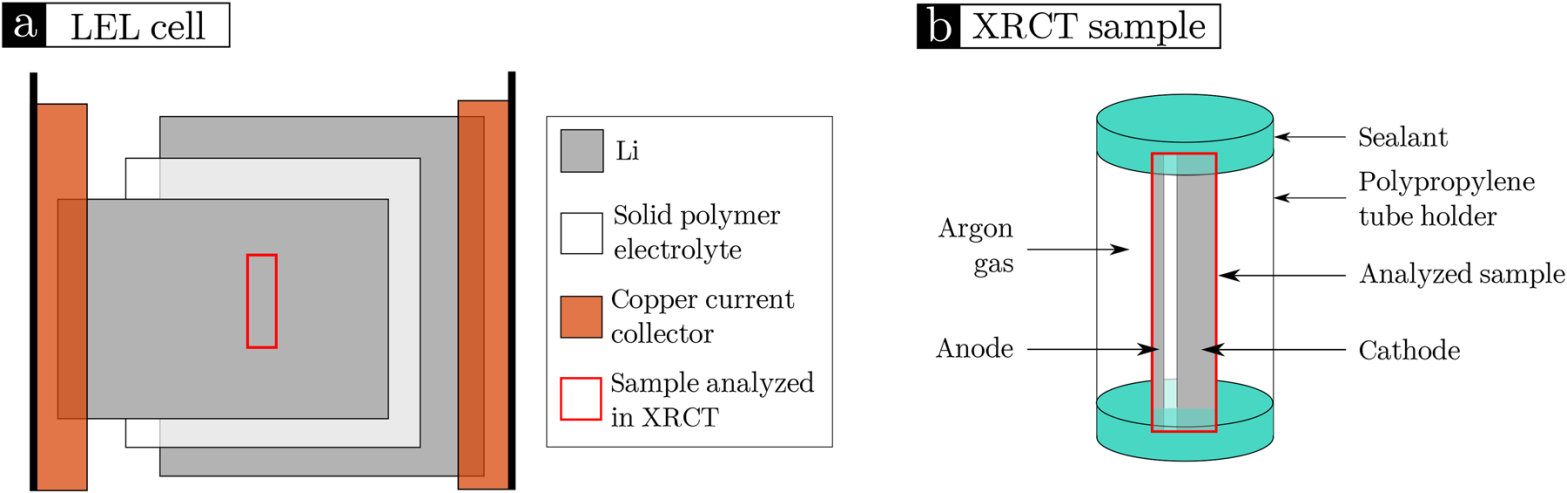
Scheme of XRCT sample preparation: (a) Li symmetric cell assembly; (b) sample sealed in a polypropylene tube.

After cycling, the cells were taken out of the holder and transferred to an argon‐filled glovebox (Jacomex) to be removed from the pouch bag. As schematized for an LEL cell in Figure 7a, a rectangle, typically of 3 mm per 10 mm, was cut at the center of the cell and placed in a 4 mm diameter polypropylene tube. Both extremities of the tube were then sealed with epoxy resin and dried for one night in the glovebox. To avoid sample motion during the acquisition, the two short edges of the cut sample were embedded within the resin sealing the tube (see Figure 7b). All the tubes were stored inside the glovebox and taken out only for X‐ray imaging purpose. For this, the samples were taken out of the glovebox and transferred to a laboratory X‐ray tomograph (EasyTom, RX Solutions). The same protocol was used for LEC and CELEC cell analyses.

#### 4.3.2 Symmetric LEL Cell Imaging Acquisition Parameters

For objects with low attenuation as typically encounter in LEL cell, a high resolution CCD camera was used in binning mode (2×2). In this mode, it was composed of 2000 rows and 1312 lines of square sensitive pixels (11.8 × 11.8 µm 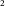). This camera had fiber optics window, which enabled high optical coupling efficiency. The fiber optics was directly coupled to CCD and a gadolinium Scintillator was coated on fiber input and it converted X‐ray image to visible image. The detector delivered the value of the attenuation with a 12 bits grey level coding resolution.

The samples were imaged at room temperature. To ensure a vertical position of the sample, the sample containing tube was glued to an alumina stem. This stem was itself fixed to the rotation platform of the tomograph. A voltage of 50 kV was used to acquire the scan with a voxel size of 1 µm. The acquisition was made with a “continuous + references” mode recording 4000 images for a 360° rotation. An exposure time of 0.60 s was used to obtain the signal required to limit noise in the acquired radiographs. The total scan time for a volume of 1880 µm × 1120 µm was 40 min. The collected radiographs were reconstructed using a filtered back projection Feldkamp‐algorithm with the commercial software provided by RX solutions to produce a 3D stack. Further image analysis was performed using the Fiji shareware [53].

#### 4.3.3 LEC and CELEC Batteries Imaging Acquisition Parameters

Because of the highly attenuating positive active material, a Paxscan 2520DX (Varex Imaging) amorphous silicon flat panel detector from Varex Imaging was used. It is composed of 1,920 rows and 1,536 lines of square sensitive pixels (127 × 127 $\mu m^{2}$) with a CsI scintillator. The detector delivers the value of the attenuation with a 16 bits grey level coding resolution. The samples were fixed to the rotation platform using the same method as for LEL samples. A voltage of 50 kV was also used to acquire the scan with a voxel size of 1 µm. The acquisition was made with a “4 pass + references” mode recording 1184 images for a 360° rotation. Each projection was the result of the average of three frames each acquired with an exposure time of 1.43 s, optimized to had good signal to noise ratio. A volume of 1800 µm × 1280 µm was imaged in 2 h. The collected radiographs were also reconstructed using the software provided by the tomograph manufacturer to produce a 3D stack and image analysis was performed with the Fiji shareware [53].

## Author Contributions

L.M. assembled and tested the cells. D.D. and R.B. assisted in the interpretation of the electrochemical data. L.M. acquired and analyzed the X‐ray tomography data. J.A. helped the acquisition of X‐ray tomography data. E.M. assisted in the interpretation and image proccessing of the tomograms. C.X. performed the simulations using GATE software. L.M., D.D., P.D, M.L., M.D., E.M., and R.B. helped discuss the results. R.B. and E.M. conceived the study. L.M., D.D., E.M., and R.B. wrote the manuscript. All the authors participated in the preparation of the manuscript.

## Conflicts of Interest

The authors declare no conflicts of interest.

## Supporting information


**Supporting File**: advs73805‐sup‐0001‐SuppMat.pdf.

## Data Availability

The data that support the findings of this study are available from the corresponding author upon reasonable request.
