# Supplementary material for: All Solid Lithium Metal‐Polymer Battery End‐of‐Life: an Investigation of Symmetric, Battery, and Bilayer Cells
Source: Adv Sci (Weinh). 2026 Mar 12;13(21):e20395. doi: 10.1002/advs.202520395 (PMC13073340; doi:10.1002/advs.202520395)
Supplement: Supplementary file 1 — Supporting File: advs73805‐sup‐0001‐SuppMat.pdf. [file ADVS-13-e20395-s001.pdf]

All Solid Lithium Metal-Polymer Battery End-of-Life: an Investigation of Symmetric, Battery and Bilayer Cells

Lucile Magnier[a,b], Didier Devaux[b], Jérôme Adrien[a], Ce Xiao[a], Philippe Dumaz[b], Margaud Lécuyer[c], Marc Deschamps[c], Eric Maire[a], Renaud Bouchet[b,*]

[a]*INSA Lyon, CNRS UMR 5510, MATEIS, 69621 Villeurbanne, France*
[b]*Univ. Grenoble Alpes, Univ. Savoie Mont Blanc, CNRS, Grenoble INP ⋆, LEPMI, 38000 Grenoble, France*
⋆ *Institute of Engineering and Management Univ. Grenoble Alpes,*
[c]*Blue Solutions, Odet, Ergué Gabéric, 29500 Quimper, France*

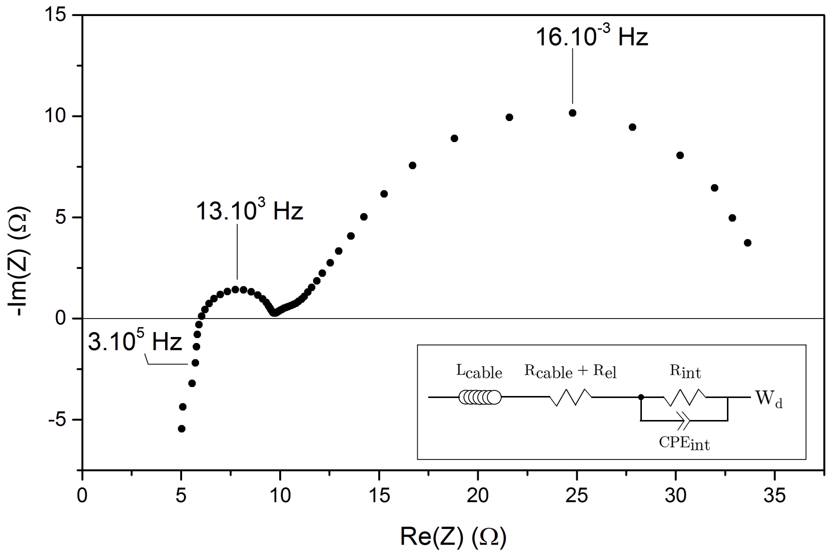

Figure S 1: Typical impedance spectrum in Nyquist representation of a LEL cell at 80°C at the initial state. The inset is a scheme of the electrical model used to fit the data.

Label	*Cycle number*
LEC#100	100
LEC#200	200
LEC#760	760
LEC#1000	1000

Table S 1: List of LEC batteries cycled at C/4, D/2. Each cell was cycled for a different cycle number.

*Corresponding author
Email address: `renaud.bouchet@grenoble-inp.fr` (Renaud Bouchet)

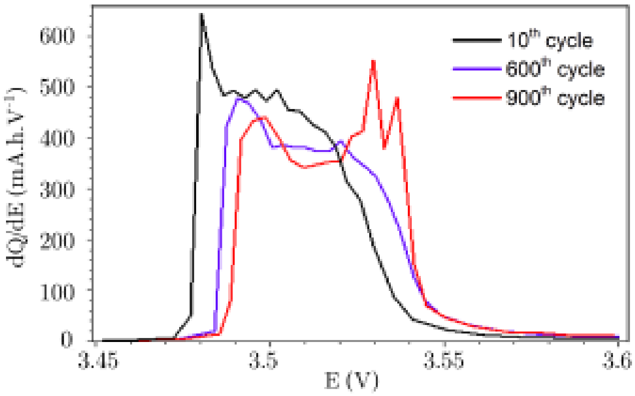

Figure S 2: Comparison of the 10th, 600th and 900th cycles of LEC#1000 battery. dQ/dE curves of capacity (Q) versus potential (E)

J $(mA.cm^{-1})$	$th_{1/2\ cycle}$ (μm)	*Cycle number*	th_1 (μm)	th_2 (μm)
0.3	48	0	0.5 then short-circuit	-
0.1	48	0	9.1 then short-circuit	-
0.05	48	0.5	48	34.4 then short-circuit
0.1	30	0.5	30	11.1 then short-circuit
0.05	30	0.5	30	20.4 then short-circuit
0.1	12	1	12	12
0.05	12	1	12	12

Table S 2: Influence on the cyclability of symmetric LEL cells of the current density (J) and the targeted Li capacity moved per half-cycle (from 9.9 $mAh.cm^{-2}$ to 2.5 $mAh.cm^{-2}$) which corresponds to a thickness $th_{1/2cycle}$. A full cycle is tested, however, the presence of a short-circuit induces a cycle number lower than 1, with th_1 the Li thickness moved in one direction and th_2 the thickness moved in the other direction. All these experiments were carried out using cells assembled with the same batch of Li and SPE.

J $(mA.cm^{-1})$	$th_{1/2\ cycle}$ (μm)	*Cycle number*
0.05	12	1/2
0.05	12	1
0.05	12	3
0.05	12	3 + 1/2
0.05	12	5
0.05	12	5 + 1/2

Table S 3: List of symmetric LEL cells cycled at current density J with the targeted Li capacity moved per half-cycle (of 2.5 $mAh.cm^{-2}$) which corresponds to a thickness $th_{1/2cycle}$. Each cell was cycled for a different cycle number. No short-circuit has occured.

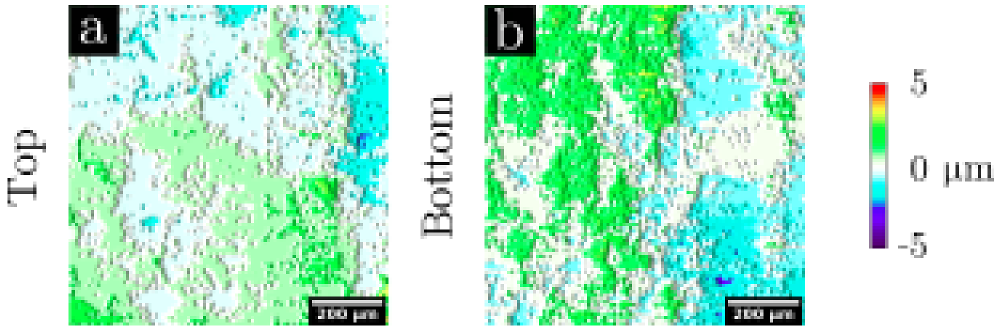

Figure S 3: 2D maps (x,y) of Li thickness variations of the Li electrode at the initial state (before cycling).

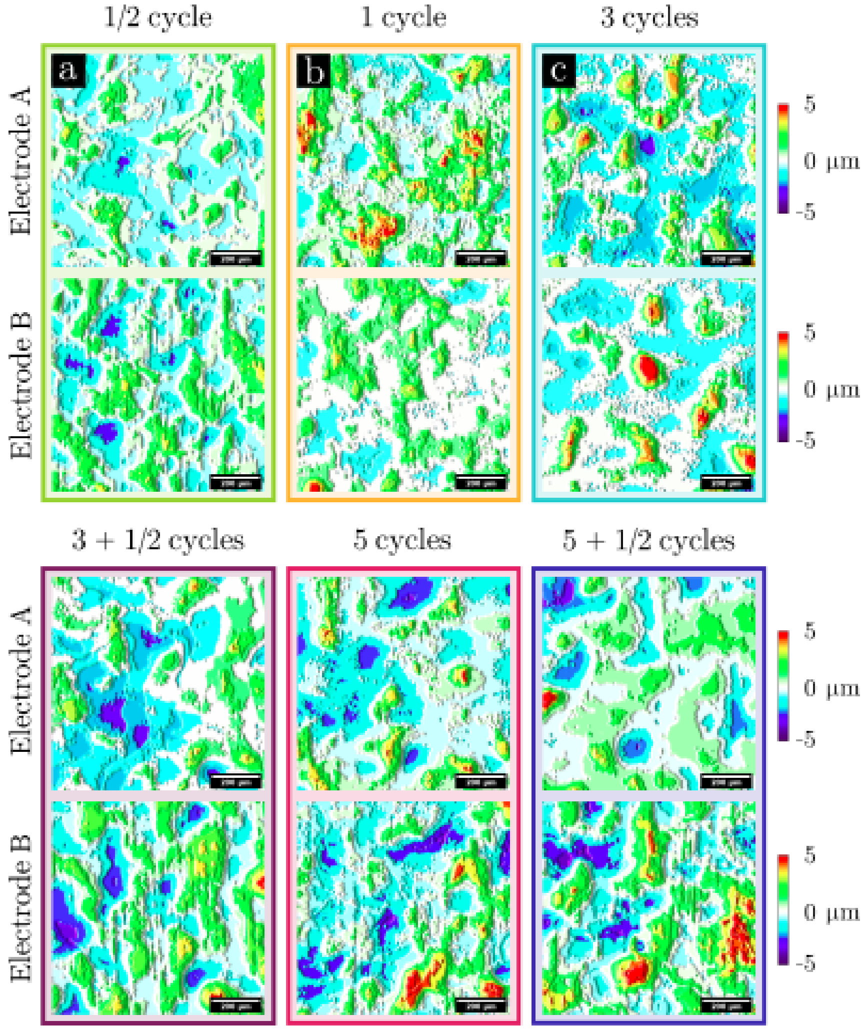

Figure S 4: 2D map (x,y) of Li thickness variations depending on the cycle number (these 900 × 900 μm^2 maps are a sub-region of the total analyzed area).

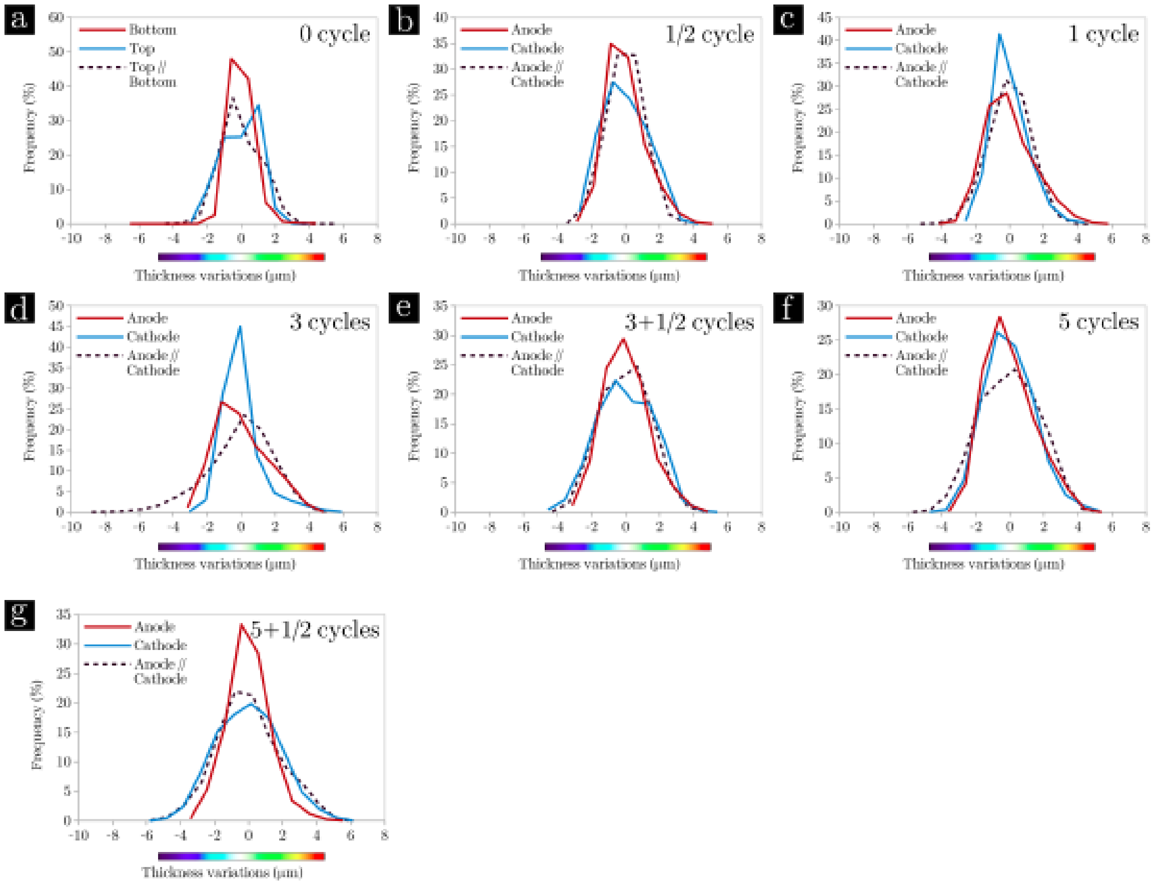

Figure S 5: Histograms extracted from the 2D maps of Figures 2.b, S3 and S4. Comparison of distributions according to the number of cycles: (a) 0 cycle, (b) ½ cycle, (c) 1 cycle, (d) 3 cycles, (e) 3 + ½ cycles, (f) 5 cycles and (g) 5 + ½ cycles.

Study of the measurement error, repeatability and representativity

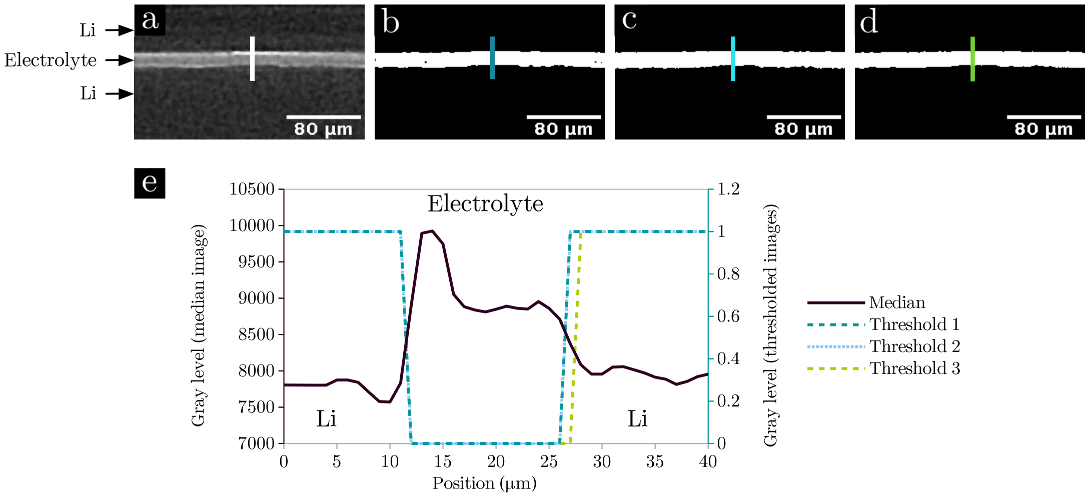

Figure S 6: Impact of thresholding on segmentation. (a) Slice of the tomographic image after median filter, (b-d) binarized images with different threshold values (b-Threshold 1, c-Threshold 2, d-Threshold 3), (e) greyscale profile along the line drawn in Figures (a-d).

The choice of the threshold grey value is made manually. Depending on the contrasts of the image and the operator, this threshold value can vary. Figure S6 illustrates the error induced during segmentation. Figure S6.a corresponds to the XRCT image before segmentation. Figures S6.b, c and d are binarized images with different threshold grey values. The quantitative comparison is shown in Figure S6.e. This graph represents the greyscale profile according to the vertical lines drawn on Figures S6.a-d. The variations induced by a change in the threshold value appear at the Li/SPE interface. At position 12 μm, the greyscale change of the "Median" profile is abrupt. The interface is then easy to segment, and its position does not vary with the threshold value. Between positions 26 and 28 μm, the greyscale transition is less sharp. The variation of the threshold value induces then an uncertainty of one voxel, *i.e.* 1 μm, on the position of the interface.

Figures S7.a, b and c are the 2D map of thickness variations of the same cell (after a 12 μm Li displacement at 0.05 $mA.cm^{-2}$) analyzed with different threshold grey values. The qualitative comparison of these maps shows that the threshold value has only a very little influence on thickness variations. The quantitative comparison, plotted in Figure S7.d, indicates a slight change in thickness distributions and the SD value is 1.37 ± 0.02 μm.

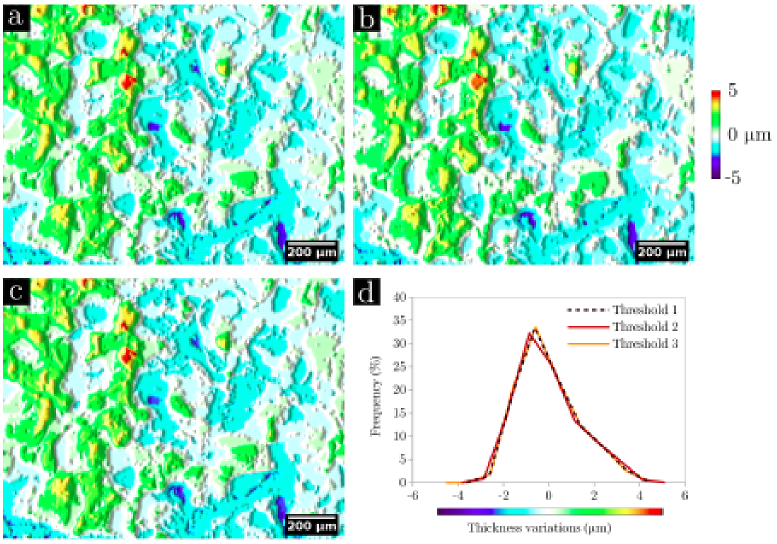

Figure S 7: Influence of the threshold value. The analyzed cell was polarized at 0.05 $mA.cm^{-2}$ to displace 12 μm of Li. 2D maps of the anode thickness variations with different threshold grey values: (a) threshold 1, (b) threshold 2 and (c) threshold 3. (d) Histograms extracted from the three maps.

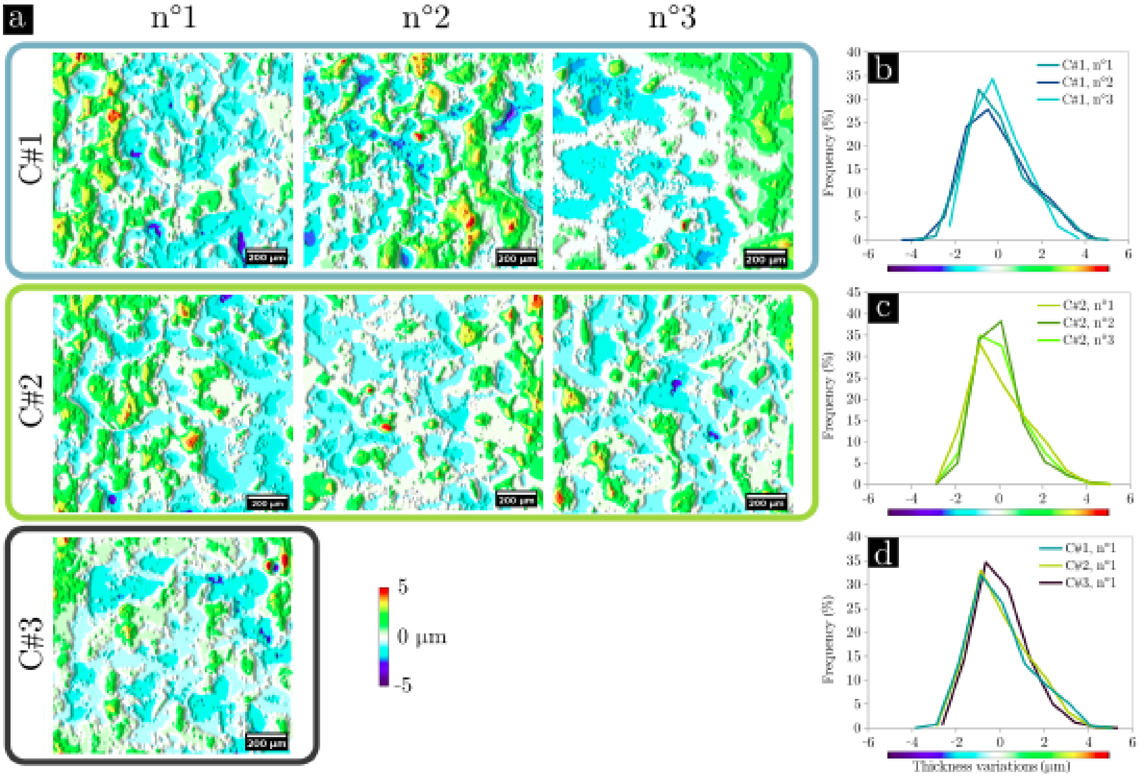

Figure S 8: (a) 2D maps of the anode thickness variations after 12 μm of Li displaced at 0.05 $mA.cm^{-2}$. Three cells were analyzed (C#1, C#2, C#3) and three areas were scanned in C#1 and C#2 cells (labelled n°1, n°2, n°3). Histograms extracted from the 2D maps of Figure S8.a: (b) comparison of the three zones analyzed in C#1 cell, (c) comparison of the three zones analyzed in C#2 cell, (d) comparison of C#1, C#2 and C#3 cells.

To validate the repeatability of the measurements, three cells (C#1, C#2 and C#3) were subjected to a 12 μm Li displacement at 0.05 $mA.cm^{-2}$. The anode thickness variation maps are shown in Figure S8.a. On two of them (C#1 and C#2), three zones in the center of the cell were characterized by XRCT (n°1, n°2, n°3). The histograms of the different zones analyzed on cells C#1 and C#2 are plotted on Figures S8.b and c, respectively. These distributions are very close from one area to another. The measurement on one area at the center of the cell is therefore representative of variations at the cell scale. The *SD* of the variations in cell C#1 is 1.3 ± 0.2 μm, and 1.2 ± 0.2 μm for cell C#2. Figure S8.c compares the distributions of zones n°1 of cells C#1, C#2 and C#3. The histograms are almost superimposed. The variations induced by a 12 μm Li displacement are thus of the same order of magnitude, whatever the analyzed cell. The *SD* of the distribution of variations after this Li displacement is 1.1 ± 0.2 μm.

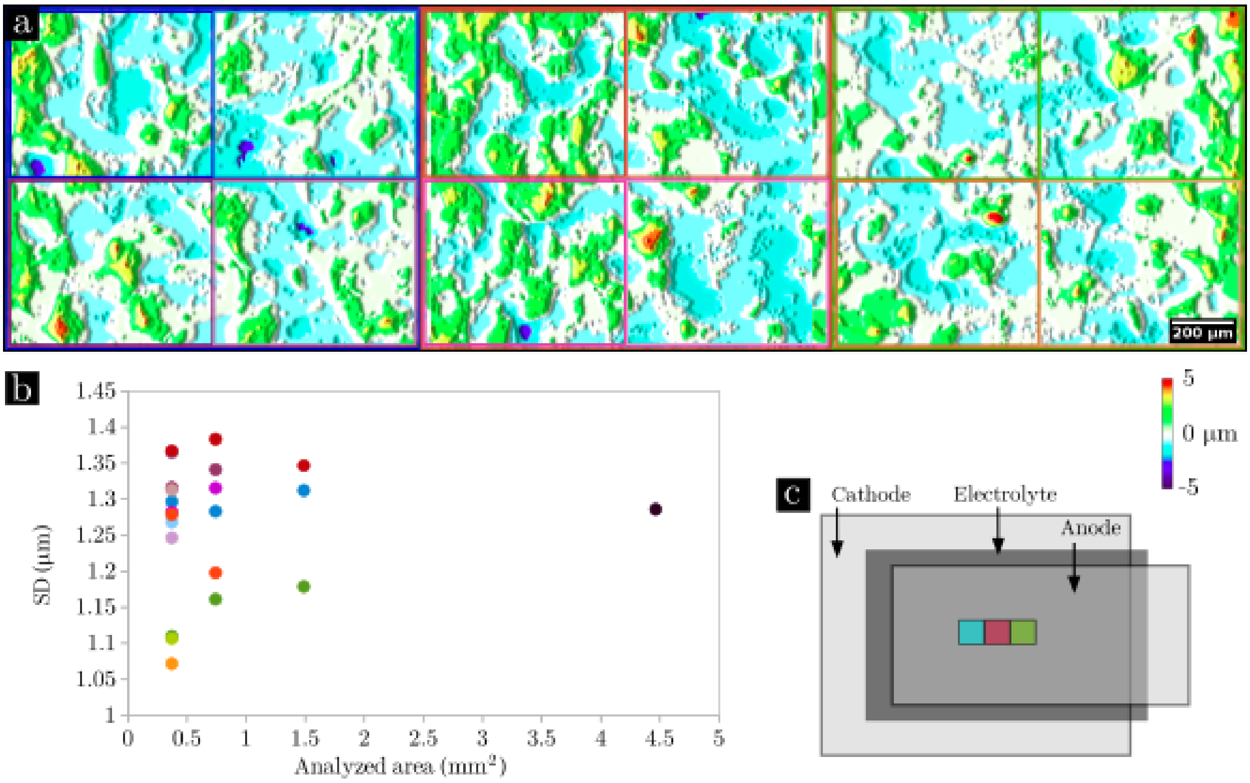

Figure S 9: Study of the representative area for the quantification of heterogeneities in a cell after moving 12 μm at 0.05 $mA.cm^{-2}$. (a) 2D map of thickness variations of a 4.5 mm^2 zone. This zone is divided into several subsections. (b) Standard deviation (*SD*) of the distributions extracted from the map according to the analyzed area.

To deepen the representativeness of a 2D map, Figure S9 compares the heterogeneities according to the analyzed area. Three scans were performed close to each other at the center of the cell, as schematized in Figure S9.c. The 2D maps calculated from these scans have an area of 1.5 mm^2. Figure S9.a represents the combination of the three maps, delimited by blue, red and green frames. The *SD* of the distributions for each zone are reported in Figure S9.b. A variation in *SD* of ± 0.2 μm is present between the three maps. The combination of these three map creates a 4.5 mm^2 map, whose *SD* is similar to the three separated maps, with the consistency of ± 0.2 μm. The division of the three maps by two and again by two, to create 0.74 mm^2 maps and 0.37 mm^2 maps, shows that *SD* value starts to fluctuate strongly from one area to the other. This demonstrates that the smaller the area, the more dispersed the SD values and the more unrepresentative it may be. An XRCT scan (leading to a map between 1.5 and 2 mm^2) is therefore representative of the behavior of a cell, with an uncertainty on *SD* of ± 0.2 μm.

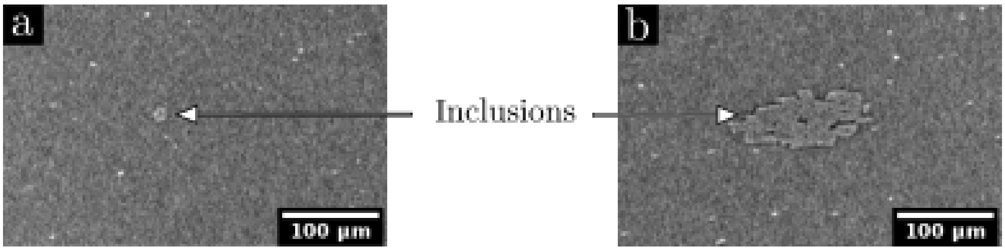

Figure S 10: XRCT slices showing the inclusions present in Li metal foils and their different shapes: (a) small inclusion, (b) very large inclusion.

	Simulated grey value
Argon	$4.84\ 10^{-10}$
Li	$5.74\ 10^{-6}$
LiH	$9.80\ 10^{-6}$
Li_3N	$2.75\ 10^{-5}$
Li_2O	$6.65\ 10^{-5}$

Table S 4: Simulation of the grey value of the different compounds simulated by the GATE software in the case of a 20 *keV* synchrotron energy source and a 0.6 *µm* voxel size. These simulations assume a monochromatic coherent source of 20 *keV* (hypothesized maximum energy in a 50 *keV* laboratory polychromatic source).

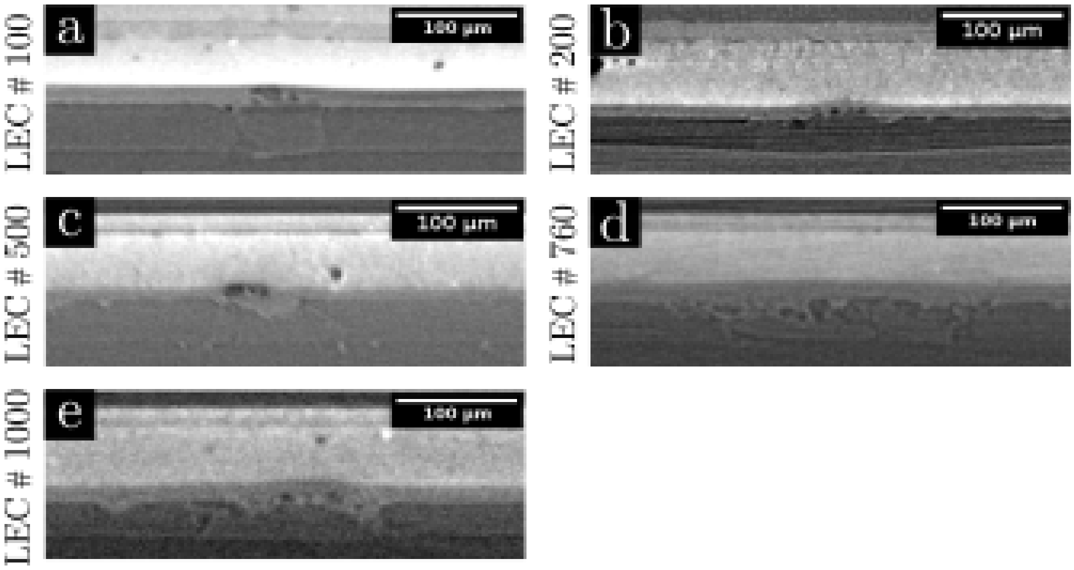

Figure S 11: Globule growth induced by the presence of inclusions in the Li metal electrode. (X,Z) slices of XRCT image of the different LEC cells: (a) LEC$#100, (b) LEC#200, (c) LEC#500, (d) LEC#760, and (e) LEC#1000.

The comparison of the impedance spectra measured at the end of the charge of the different cycles of LEC$#1000 is given in Figure S12.a. Due to the large inductive effect of the cables compared to the low impedance of the cell, it was not possible to fully separate the different contributions in the spectra. However, the low frequency contribution due to the intercalation processes in the $LiFePO_4$ active materials is not impacted while a drift to larger resistance of the high frequency contribution is observed. Thus, it seems that most probably the electrolyte contribution and the Li/electrolyte interface are impacted. This is confirmed by the subtraction between the 600th and 900th cycles spectrum and the 10th cycle spectrum that are reported in Figure S12.b. In this graph, we observe only what has been changed along cycling. Below 100 Hz which corresponds to the answer of the positive electrode with charge transfer contribution, diffusion in the thickness of the electrode (tortuosity) and solid-state diffusion in LiFePO4,[1] there is absolutely no contribution which confirms that in average the positive electrode contribution remains unchanged during cycling. However, we observe a shift of the high frequency resistance to higher value and the appearance of a small loop around 8 kHz. It suggests a degradation of the Li/polymer interface, with possibly the loss of contact locally at the Li/electrolyte interface which will impact both the electrolyte and SEI resistances.

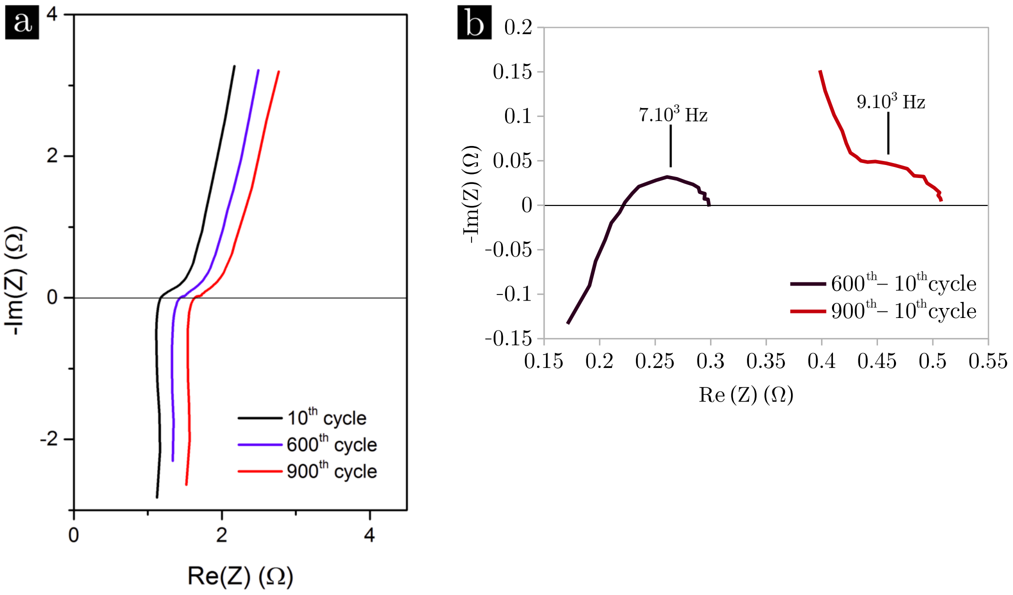

Figure S 12: (a) Impedance spectra in Nyquist representation of LEC$#1000 battery. These spectra are acquired after the charge. (b) Subtraction of the spectra of the 900th and 10th cycles and of the 600th and 10th cycles.

References

[1] D. Devaux, H. Leduc, P. Dumaz, M. Lecuyer, M. Deschamps, R. Bouchet, Effect of Electrode and Electrolyte Thicknesses on All-Solid-State Battery Performance Analyzed With the Sand Equation, Frontiers in Energy Research 7 (2020) 168. doi:10.3389/fenrg.2019.00168. URL `https://www.frontiersin.org/article/10.3389/fenrg.2019.00168/full`
